# Reversing the Interfacial Electric Field in Metal Phosphide Heterojunction by Fe‐Doping for Large‐Current Oxygen Evolution Reaction

**DOI:** 10.1002/advs.202308477

**Published:** 2024-04-08

**Authors:** Zhong Li, Chengshuang Xu, Zheye Zhang, Shan Xia, Dongsheng Li, Liren Liu, Peng Chen, Xiaochen Dong

**Affiliations:** ^1^ Center for Rehabilitation Medicine, Rehabilitation & Sports Medicine Research Institute of Zhejiang Province, Department of Rehabilitation Medicine, Cancer Center, Zhejiang Provincial People's Hospital (Affiliated People's Hospital) Hangzhou Medical College Hangzhou Zhejiang 310014 China; ^2^ Key Laboratory of Flexible Electronics (KLOFE) & Institute of Advanced Materials (IAM), School of Flexible Electronics (Future Technologies), School of Physical and Mathematical Sciences Nanjing Tech University (NanjingTech) Nanjing 211816 China; ^3^ School of Chemistry, Chemical Engineering and Biotechnology, Institute for Digital Molecular Analytics and Science Nanyang Technological University Singapore 637457 Singapore; ^4^ College of Materials and Chemical Engineering, Key Laboratory of Inorganic Nonmetallic Crystalline and Energy Conversion Materials China Three Gorges University Yichang 443002 China

**Keywords:** HER, heterojunction catalysts, interfacial engineering, OER, water splitting

## Abstract

Developing non‐precious‐metal electrocatalysts that can operate with a low overpotential at a high current density for industrial application is challenging. Heterogeneous bimetallic phosphides have attracted much interest. Despite high hydrogen evolution reaction (HER) performance, the ordinary oxygen evolution reaction (OER) performance hinders their practical use. Herein, it is shown that Fe‐doping reverses and enlarges the interfacial electrical field at the heterojunction, turning the H intermediate favorable binding sites for HER into O intermediate favorable sites for OER. Specifically, the self‐supported heterojunction catalysts on nickel foam (CoP@Ni_2_P/NF and Fe‐CoP@Fe‐Ni_2_P/NF) are readily synthesized. They only require the overpotentials of 266 and 274 mV to drive a large current density of 1000 mA cm^−2^ (*j*_1000_) for HER and OER, respectively. Furthermore, a water splitting cell equipped with these electrodes only requires a voltage of 1.724 V to drive *j*_1000_ with excellent durability, demonstrating the potential of industrial application. This work offers new insights on interfacial engineering for heterojunction catalysts.

## Graphical abstract

A new interfacial engineering strategy is utilized to modulate the catalytic properties of self‐supported heterojunction catalysts by Fe doping. It can reverse and enlarge the interfacial electric field, thereby converting the HER‐active sites in CoP@Ni_2_P/NF to OER‐active stie in Fe‐CoP@Fe‐Ni_2_P/NF. The overall water splitting cell equipped with the two electrodes only required a low voltage of 1.724 V to drive the *j*_1000_ with long‐term stability.

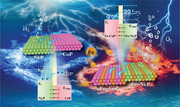

## 1 Introduction

Hydrogen energy generated by electrochemical water splitting is hurdled by the reliance on the precious metal‐based electrocatalysts (e.g., Pt for HER and IrO_2_/RuO_2_ for OER), and the slow kinetics of oxygen evolution reaction (OER) at the anode.^[^ ^1^, ^2^, ^3^ ^]^ Although tremendous effort has been made to develop various non‐precious‐metal catalysts, the water‐splitting cells constructed by these catalysts often suffer from high driving voltage (>1.8 V) or poor stability at a large current density,^[^ ^4^, ^5^ ^]^ falling short to the requirement for industrial application.

To enhance the current density as a given voltage by enlarging the electroactive surface area, synthesized electrocatalysts are deposited onto 3D electrodes, such as Ni, Co, and Fe foams.^[^ ^6^, ^7^, ^8^ ^]^ In situ growth of electrocatalysts onto these self‐supported electrodes without the need of binders simplifies the synthesis and improves the charge transfer and stability.^[^ ^9^, ^10^, ^11^, ^12^ ^]^ But in situ growth of rationally‐designed high‐performance catalysts is challenging.

Transition metal phosphides (TMPs) have attracted significant attention due to their better conductivity comparing to metal oxides, high HER catalytic activity, and low cost.^[^ ^13^, ^14^, ^15^ ^]^ Bimetallic phosphides with heterojunction exhibit improved HER performance because the established interfacial electric field facilitates electron migration, resulting in negatively charged active sites favorable for the adsorption of hydrogen intermediate.^[^ ^16^, ^17^ ^]^ On the other hand, the ordinary OER performance of metal phosphide further deteriorates because the negatively charged active sites are unfavorable for adsorption of oxygen intermediates.^[^ ^18^, ^19^, ^20^ ^]^ To address this issue, researchers replace one phase in the bimetallic phosphide heterojunction with an OER active phase (e.g., metal oxides, metal (oxy)hydroxide) which has a lower work function (Φ) than metal phosphides to create OER/HER bifunctional catalysts.^[^ ^21^, ^22^ ^]^ But the fabrication process is sophisticated (thus costly). In addition, coupling TMP with oxides or (oxy)hydroxides causes electron delocalization at the metal sites, hindering electron transfer at the interface of heterojunction.^[^ ^23^ ^]^ We conceive that OER performance of TMP heterojunction catalysts may be improved by establishing appropriate interfacial electric field and widening the work function difference between the two phases to create electron‐deficient active site and promote electron transfer simply through heteroatom doping.

Herein, we grew transition metal phosphide heterojunction (CoP@Ni_2_P) on Ni foam (NF) which only requires a small overpotential of 266 mV to drive the large current density of ‐1000 mA cm^−2^ (*j*_‐1000_) for HER. And an OER electrode (Fe‐CoP@Fe‐Ni_2_P/NF) was constructed by doping Fe into CoP@Ni_2_P, which only requires a small overpotential of 274 mV to drive *j*_1000_. Experimental and density functional theory (DFT) results reveal that Fe dopant reverses and enlarges the interfacial electric field, turning the electron‐rich Ni sites in CoP@Ni_2_P/NF into electron‐deficient to facilitate adsorption of oxygen intermediates. The overall water splitting cell equipped with these self‐supported electrodes can drive *j*_1000_ at a low voltage of 1.724 V with excellent long‐term stability. This study proposes a new strategy of interfacial engineering for heterojunction catalysts, specifically, modulating the interfacial electric field by doping metallic elements.

## 2 Results and Discussion

### 2.1 Synthesis of Electrocatalysts

**Scheme 1:**
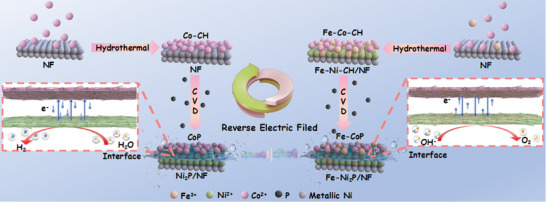
Illustration for synthesis of CoP@Ni_2_P/NF and Fe‐CoP@Fe‐Ni_2_P/NF heterogeneous catalysts.

CoP@Ni_2_P/NF and Fe‐CoP@Fe‐Ni_2_P/NF were synthesized via a two‐step process involving hydrothermal treatment and subsequent phosphorization (**Scheme** **1**). In the first step, the reaction temperature is lower than the typical hydrothermal condition (90 °C vs >100 °C) in order to slow down the reaction rate whereby leaving some surface of NF for phosphorization. On the other hand, it is sufficient to decompose urea for the growth of cobalt carbonate hydroxide on NF (Co‐CH/NF):^[^ ^24^ ^]^

(1) $$NH_{2}-\left(C\;=\;O\right)-NH_{2}+H_{2}O\;\to \;2NH_{3}+CO_{2}$$

(2) $$CO_{2}+H_{2}O\;\to \;C{O_{3}}^{2-}+2H^{+}$$

(3) $$NH_{3}+\;H_{2}O\;\to \;N{H_{4}}^{+}+\;OH^{-}$$

(4) $$Co^{2+}\;+\;OH^{-}\;+\;C{O_{3}}^{2-}\;\to \;\mathrm{Co}-\mathrm{CH}$$

If Fe^3+^ is added into the reaction, it corrodes NF and form a Fe‐Ni‐CH layer on the surface:^[^ ^25^ ^]^

(5) $$2Fe^{3+}+\mathrm{Ni}\to 2Fe^{2+}+Ni^{2+}$$

(6) $$4Fe^{2+}+\;2H_{2}O\;+\;O_{2}\to \;4\;Fe^{3+}+4\;OH^{-}$$

(7) $$Ni^{2+}\;+\;Fe^{3+}+\;OH^{-}\;+\;C{O_{3}}^{2-}\;\to \;\mathrm{Fe}-\mathrm{Ni}-\mathrm{CH}$$

Co^2+^ and Fe^3+^ ions then precipitate onto the corroded NF surface, to obtain Fe‐Co‐CH@Fe‐Ni‐CH/NF:

(8) $$Co^{2+}+\;Fe^{3+}+\;OH^{-}+\;C{O_{3}}^{2-}\to \;\mathrm{Fe}-\mathrm{Co}-\mathrm{CH}$$

The second phosphorization step is realized by chemical vapor deposition (CVD). PH_3_ gas, generated from decomposition of NaH_2_PO_2_·H_2_O at 300 °C, simultaneously phosphorizes Co‐CH and exposed NF surface in Co‐CH/NF or Fe‐Co‐CH and Fe‐Ni‐CH in Fe‐Co‐CH@Fe‐Ni‐CH/NF, producing two self‐supported electrodes with heterostructures (CoP@Ni_2_P/NF as cathode and Fe‐CoP@Fe‐Ni_2_P/NF as anode).

### 2.2 Characterizations

We first confirmed that metallic NF can be phosphorized to Ni_2_P/NF. As uncovered by the scanning electron microscopy (SEM), the surface of NF (Figure S1a, Supporting Information) became rough after phosphorization (Figure S1b, Supporting Information). The energy dispersive X‐ray spectroscopy (EDX) mapping shows uniform distribution of Ni and P (Figure S1c, Supporting Information). And X‐ray diffraction (XRD) pattern exhibits three characteristic peaks at 40.1, 47.8, and 54.5° corresponding to the (1 1 1), (2 1 0), and (3 0 0) facets of Ni_2_P (PDF#03‐0953) (Figure S2, Supporting Information).

**Figure 1:**
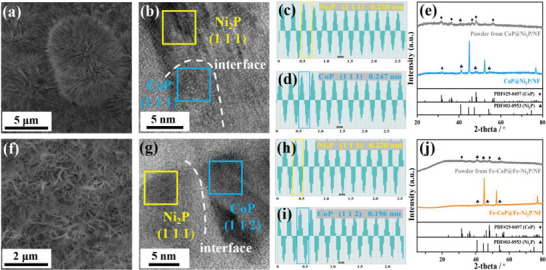
Characterizations of a–e) CoP@Ni_2_P/NF and f–j) Fe‐CoP@Fe‐Ni_2_P/NF. a,f) SEM images. b,g) HRTEM images (dash line indicates the interface between Ni_2_P and CoP). (c and d; h and i) Line scan of inverse fast Fourier transform (FFT) of facets of Ni_2_P and facets of CoP. e,j) XRD patterns.

For CoP@Ni_2_P/NF, needle‐shaped CoP is uniformly distributed on the surface of NF (**Figure** **1**a). EDX mapping of CoP@Ni_2_P/NF cross‐section (Figure S3a, Supporting Information) reveals P doping into NF skeleton, forming a thin layer of Ni_2_P/NF. The uniform distribution of both Co and P above Ni_2_P/NF indicates the formation of a heterojunction between Co phosphide and Ni phosphide. The high‐resolution transmission electron microscopy (HRTEM) image further shows the interface between the two metal phosphides and reveals lattice fringe spacings of 0.220 and 0.247 nm corresponding to the (1 1 1) facets of Ni_2_P and CoP, respectively (Figure 1b–d). The XRD pattern (Figure 1e) of CoP@Ni_2_P/NF exhibits three characteristic peaks of Ni_2_P, similar to Ni_2_P/NF. The powders obtained by sonicating CoP@Ni_2_P/NF give peaks at 31.5, 36.3, 46.2, 48.1, and 56.2° corresponding to the (0 1 1), (1 1 1), (1 1 2), (2 1 1), and (0 2 0) facets of CoP. These results confirm the heterostructure of CoP@Ni_2_P/NF.

In contrast to the needle‐shaped CoP, Fe‐CoP@Fe‐Ni_2_P heterojunction is sheet‐shaped due to the change of crystal structure upon incorporation of high valence Fe^3+^ (Figure 1f). As shown in EDX mapping of Fe‐CoP@Fe‐Ni_2_P/NF cross section (Figure S3b, Supporting Information), Co, Ni, Fe, P are uniformly dispersed on the NF. Moreover, the TEM mapping of Fe‐CoP@Fe‐Ni_2_P powder peeled off from NF by ultrasound treatment also shows an uniformly distribution of Fe in CoP and Ni_2_P phases (Figure S4, Supporting Information), indicating that Fe is simultaneously doped into CoP and Ni_2_P phases. The HRTEM image of Fe‐CoP@Fe‐Ni_2_P/NF reveals the interface between CoP and Ni_2_P, with lattice fringes corresponding to the (1 1 1) and (1 1 2) facets of Ni_2_P and CoP, respectively (Figure 1g). No evidence suggests the existence of Fe‐based phosphide (Figure 1h,i), confirming that Fe exists as the dopant in Fe‐CoP@Fe‐Ni_2_P/NF. The XRD patterns of powders obtained by sonication from Fe‐CoP@Fe‐Ni_2_P/NF and self‐supported Fe‐CoP@Fe‐Ni_2_P/NF only exhibit the peak of CoP and Ni_2_P (Figure 1j), consistent with the HRTEM observations.

### 2.3 HER Performance of Electrocatalysts

**Figure 2:**
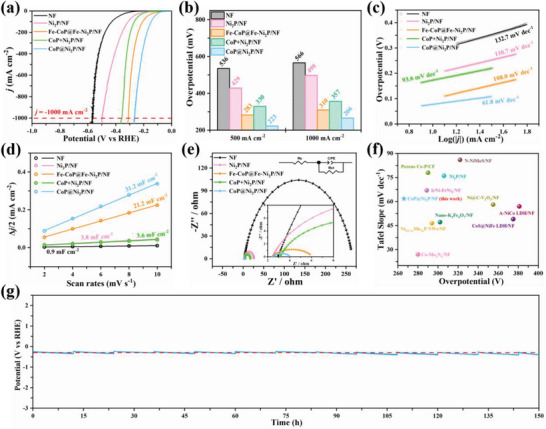
HER activities of samples: a) Polarization curves. b) Overpotentials at *j_‐_*_500_ and *j_‐_*_1000_. c) Tafel plots. d) Double‐layer capacitances (C_dl_). e) EIS curves (Insert shows the equivalent circuit). f) Comparison of the Tafel slopes and overpotentials with other self‐supporting electrocatalysts at *j_‐_*_1000_. g) The chronopotentiometric curve of CoP@Ni_2_P/NF at *j_‐_*_1000_ for 150 h (Replace the electrolyte regularly, with a interval of every 12 h).

We evaluated the HER performance of the samples using a three‐electrode system in a 1 m KOH solution at room temperature. As shown in **Figure** **2**a, CoP@Ni_2_P/NF shows the highest HER performance, with a low overpotential of 266 mV to achieve *j*_‐1000_, which is much lower than pure NF, Ni_2_P/NF, and Fe‐CoP@Fe‐Ni_2_P/NF (Figure 2b). In addition, CoP@Ni_2_P/NF demonstrates the lowest Tafel slope of 61.8 mV dec^−1^ (Figure 2c), indicating fast HER kinetics and a Volmer–Heyrovsky electrocatalytic process. Double‐layer capacitance (C_dl_) was calculated from cyclic voltammetry (CV) in the non‐Faradaic region (Figure S5, Supporting Information). CoP@Ni_2_P/NF shows the highest C_dl_ of 31.2 mF cm^−2^ (Figure 2d), indicating the largest active area. Furthermore, the charge‐transfer resistance (R_ct_) was determined by fitting electrochemical impedance spectroscopy (EIS) data (Figure 2e; Table S1, Supporting Information). CoP@Ni_2_P/NF exhibits the lowest R_ct_ (1.5 Ω), indicating a fast charge transfer rate. In comparison, the mechanical mixture of CoP and Ni_2_P/NF has a higher overpotential (357 mV) at *j*_‐1000_, a higher Tafel slope (93.6 mV dec^−1^), a lower C_dl_ (3.6 mF cm^−2^), and a higher R_ct_ (14.6 Ω) than CoP@Ni_2_P/NF, suggesting the in situ grown heterostructure is the key to the superior catalytic activity. Altogether, the excellent HER performance of CoP@Ni_2_P/NF is attributed to its high intrinsic catalytic activity, fast OER kinetics, and low charge transfer resistance, which outperforms previously reported self‐supporting electrocatalysts (Figure 2f; Table S2, Supporting Information).

To evaluate the long‐term stability of CoP@Ni_2_P/NF, chronopotentiometric measurement at *j*_‐1000_ was conducted. As shown in Figure 2g, the overpotential of CoP@Ni_2_P/NF did not increase significantly after 150 h of testing. Although the surface of the catalyst became rough after the long‐term test, the characteristic peaks in the XRD pattern assigned to metal phosphides did not shift, and no new peaks appeared in the Raman spectrum (Figure S6a–c, Supporting Information), indicating that no new species were generated during HER. The additional small peaks in the XRD pattern of powders from CoP@Ni_2_P/NF are assigned to Co(OH)_2_. It has been shown that CoP can be slightly oxidized in KOH electrolyte into HER‐inactive Co(OH)_2_.^[^ ^26^, ^27^ ^]^ The interface between CoP and Ni_2_P phases for CoP@Ni_2_P was well preserved after long‐term HER test, with the lattice fringe spacings of 0.220 and 0.190 nm corresponding to the (1 1 1) and (2 1 1) facets of Ni_2_P and CoP, respectively (Figure S6d–f, Supporting Information). In addition, X‐ray photoelectron spectroscopy (XPS) spectrum remained unaltered, except that the peak intensity of metal phosphides was slightly reduced (Figure S7, Supporting Information). Taken together, the CoP@Ni_2_P/NF shows good long‐term stability at a high current density and thus is a suitable cathode for industrial water‐splitting applications.

### 2.4 OER Performance of Electrocatalysts

**Figure 3:**
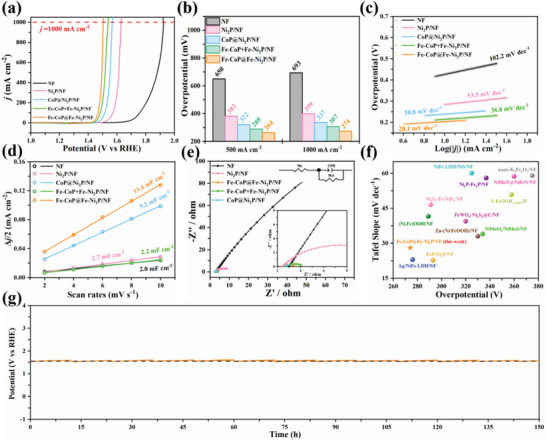
OER activities of samples. a) Polarization curves. b) Overpotentials at *j*_500_ and *j*_1000_. c) Tafel plots. d) Double‐layer capacitances (C_dl_). e) EIS curves (Insert shows the equivalent circuit). f) Comparison of the Tafel slopes and overpotentials with other self‐supporting electrocatalysts at *j*_1000_. g) The chronopotentiometric curve of Fe‐CoP@Fe‐Ni_2_P/NF at *j*_1000_ for 150 h (Replace the electrolyte regularly, with a interval of every 12 h).

**Figure** **3**a shows the OER performance of the electrocatalysts. Among them, Fe‐CoP@Fe‐Ni_2_P/NF exhibits superior OER activity (274 mV) compared to CoP@Ni_2_P/NF (337 mV) at *j*_1000_ (Figure 3b). Notably, further increasing the molar ratio of Fe/Co precursor during the synthesis process, the overpotential of Fe‐doping heterojunction (Fe‐CoP@Fe‐Ni_2_P/NF) is even slightly increased (Figure S8, Supporting Information), likely because excess doping into metal phosphide adversely affects the electron transfer^[^ ^4^, ^28^ ^]^ and the NF is over‐etched by Fe ions. The Tafel slope of Fe‐CoP@Fe‐Ni_2_P/NF is 28.1 mV dec^−1^, indicating fast OER kinetics, and that the rate‐determining step (RDS) is the conversion of O* to OOH* (Figure 3c).^[^ ^29^, ^30^ ^]^ Moreover, Fe‐CoP@Fe‐Ni_2_P/NF has a higher C_dl_ (11.6 mF cm^−2^) (Figure 3d; Figure S9, Supporting Information) and lower R_ct_ (1.1 Ω) (Figure 3e; Table S3, Supporting Information) than other samples, implying a larger active surface area and faster charge transfer. Notably, the mechanical mixture of Fe‐CoP and Fe‐Ni_2_P/NF exhibits a higher overpotential (307 mV) at *j*_1000_, higher Tafel slope (34.8 mV dec^−1^), lower C_dl_ (2.2 mF cm^−2^), and higher R_ct_ (1.6 Ω) than Fe‐CoP@Fe‐Ni_2_P/NF, testifying the importance of in situ grown heterojunction. In sum, the excellent OER performance of Fe‐CoP@Fe‐Ni_2_P/NF is due to its high intrinsic catalytic activity, fast OER kinetics, and low charge transfer resistance, which surpasses that of previously reported self‐supporting electrocatalysts (Figure 3f; Table S4, Supporting Information).

As shown in Figure 3g and Table S4, Supporting Information), compared to recent reported electrocatalysts, the overpotential of Fe‐CoP@Fe‐Ni_2_P/NF only increased by 20 mV after 150 h chronopotentiometry, showcasing a good long‐term stability. The catalyst surface became only a little rougher after long‐term test (Figure S10a, Supporting Information). As the standard enthalpies (ΔH_f_^0^) and Gibbs energies (ΔG_f_^0^) of metal (oxy)hydroxides are more negative than that of metal phosphides,^[^ ^31^, ^32^ ^]^ Fe‐CoP@Fe‐Ni_2_P should be oxidized under OER condition. Indeed, after long‐term OER, the characteristic XRD peaks of metal phosphides nearly vanished (Figure S10b, Supporting Information), a new broad Raman peak between 400 and 600 cm^−1^ attributable to Co/Ni (oxy)hydroxides appeared (Figure S10c, Supporting Information).^[^ ^33^, ^34^, ^35^ ^]^ The characteristic peak of FeOOH at 670 cm^−1^ was not observed,^[^ ^36^, ^37^ ^]^ indicating the Fe still existed in doping form during the reconstruction process. Moreover, the peak corresponding to P element was not observed from XPS survey spectrum of Fe‐CoP@Fe‐Ni_2_P/NF after OER test (Figure S11, Supporting Information), indicating that P was leached into the solution during OER. As shown in Figure S10d–h (Supporting Information), the HRTEM image of Fe‐CoP@Fe‐Ni_2_P/NF after long‐term OER test showed a clear interface between Fe‐CoOOH and Fe‐NiOOH. The lattice fringe spacing of 0.239 nm is corresponding to the (0 1 1) facet of NiOOH (Refer to PDF#00‐027‐0956), whereas the lattice fringe spacings of 0.243, 0.230, 0.235 nm are corresponding to the (0 2 1), (1 1 1), (0 4 0) facets of CoOOH (Refer to PDF#00‐026‐0480). Moreover, as shown in Figure S12a (Supporting Information), for a fresh Fe‐CoP@Fe‐Ni_2_P/NF catalyst, when the potential increased from 1 to 1.8 V (V vs RHE) in the first CV cycle, the current density is always higher than that of the subsequent cycles, likely because of the contribution from the irreversible transformation of the metal phosphide catalyst into metal hydroxide. Similar phenomenon was observed in the previous studies.^[^ ^38^, ^39^ ^]^ At high potential, the metal hydroxide will be further reversibly transformed into metal (oxy)hydroxide. To verify it, we further measured the CV of Fe‐CoP@Fe‐Ni_2_P after 10 cycle CV test with a low scan rate (Figure S12b, Supporting Information). When the potential increased from 1 to 1.8 V (V vs RHE), an obvious oxidation peak around 1.3–1.5 V versus RHE was observed, due to oxidation of Co^2+^ and Ni^2+^ to Co^3+^ and Ni^3+^. When the potential decreased from 1.8 to 1 V (V vs RHE) to complete a CV cycle, a reduction peak could be observed at low potential (1.1–1.3 V vs RHE), indicating that Co^3+^ and Ni^3+^ were reduced back to Co^2+^ and Ni^2+^. This reversible transformation is in accordance with the previous reports.^[^ ^40^, ^41^ ^]^ Considering that OER is performed at a high potential, the actual active phase is metal (oxy)hydroxide (Fe‐CoOOH@Fe‐NiOOH). During this structural reconstruction process, unsaturated metal sites might be in situ generated,^[^ ^31^, ^42^ ^]^ and the loss of cation or anion species would lead to formation of low crystallinity or even amorphous phases, thus increasing surface area and exposure of active sites,^[^ ^43^ ^]^ which can enhance OER performance. These results suggest that Fe‐CoP@Fe‐Ni_2_P/NF (actually, Fe‐CoOOH@Fe‐NiOOH/NF) offers good long‐term stability at a high current density and thus is a suitable anode for industrial water‐splitting applications.

### 2.5 Catalytic Mechanisms

**Figure 4:**
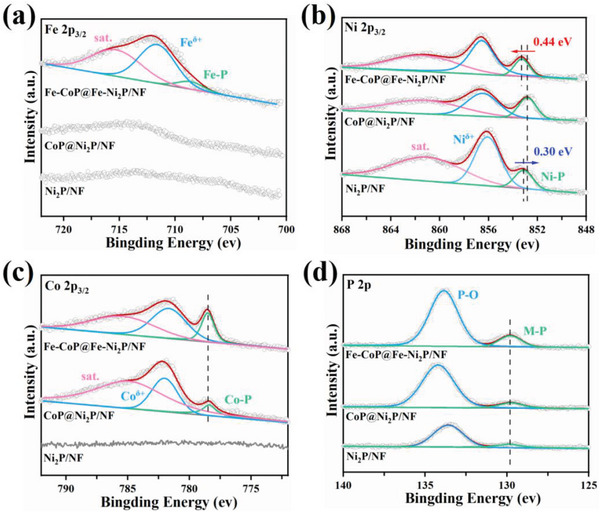
XPS spectra of Ni_2_P/NF, CoP@Ni_2_P/NF, and Fe‐CoP@Fe‐Ni_2_P/NF. a) Fe 2p_3/2_. b) Ni 2p_3/2_. c) Co 2p_3/2_. d) P 2p.

The catalytic mechanisms of CoP@Ni_2_P/NF and Fe‐CoP@Fe‐Ni_2_P/NF for HER and OER were investigated using characterizations and density functional theory (DFT) calculations. X‐ray photoelectron spectroscopy (XPS) (**Figure** **4**a) shows that Fe is successfully doped into the metal phosphide heterojunction, as indicated by the binding energy (BE) at 708.6 eV arising from Fe─P bond in Fe‐CoP@Fe‐Ni_2_P/NF.^[^ ^44^ ^]^ For Ni_2_P/NF, the peak at 853.12 eV is resulted from Ni‐P bond, while the BE at 856.08 eV corresponds to Ni^δ+^ due to the surface oxidation (Figure 4b).^[^ ^45^, ^46^ ^]^ For CoP@Ni_2_P/NF with the heterostructure, the BE of Ni‐P down‐shifts to 852.82 eV, indicating an increased negative charge of Ni in Ni─P bond due to electron transfer at the interface. The electron‐accepting Ni site favors the adsorption of intermediate H*, which is desirable for HER.^[^ ^47^ ^]^ In contrast, the BE of Ni─P in Fe‐CoP@Fe‐Ni_2_P/NF up‐shifts to 853.26 eV as compared to that in CoP@Ni_2_P/NF, indicating that Ni is deprived of electrons by Fe doping. The resulting positive charge environment around Ni site in Ni─P facilities the adsorption of O intermediates during OER.^[^ ^48^ ^]^ For Fe‐CoP@Fe‐Ni_2_P/NF and CoP@Ni_2_P/NF, the BE at 778.38 eV is originated from Co in Co─P bonds (Figure 4c), and BE at 129.85 eV is attributed by P in metal‐P bonds in all the three samples (Figure 4d).^[^ ^49^, ^50^ ^]^ No significant shift of the two peaks is observed after Fe doping, indicating that Fe doping mainly affects the charge density around Ni site in Ni─P.

**Figure 5:**
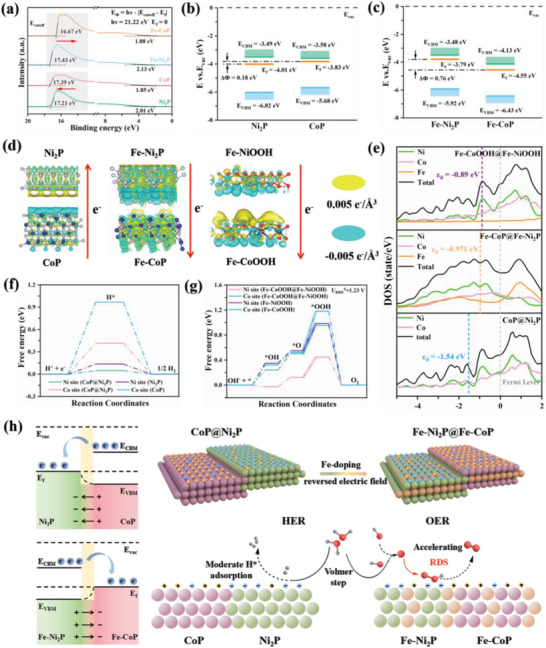
Mechanistic studies. a) UPS spectra of Ni_2_P, CoP, Fe‐Ni_2_P, and Fe‐CoP. b) Energy band diagrams of Ni_2_P and CoP (E_vac_: Vacuum level. E_CBM_: Conduction band minimum. E_f_: Fermi level. E_VBM_: Valence band maximum). c) Energy band diagrams of Fe‐Ni_2_P and Fe‐CoP. d) Charge density differences at the interface of heterojunctions (CoP@Ni_2_P, Fe‐CoP@Fe‐Ni_2_P and Fe‐CoOOH@Fe‐NiOOH). e) Density of states (DOS). f) Free energy diagrams of Ni_2_P, CoP and CoP@Ni_2_P for HER. g) Free energy diagrams of Fe‐NiOOH, Fe‐CoOOH, and Fe‐NiOOH@Fe‐CoOOH for OER. h) Illustration of the proposed mechanism.

When two components with different work functions (Φ, defined as the energy required for an electron located at the Fermi level moving to the vacuum level) are brought together to form a heterojunction, an electric field will be established at the interface whereby causing transfer of electrons from the lower Φ side to the higher side.^[^ ^51^, ^52^ ^]^ Based on ultraviolet photoelectron spectroscopy (UPS) (**Figure** **5**a), the estimated Φs of Ni_2_P, CoP, Fe‐Ni_2_P and Fe‐CoP are 4.01, 3.83, 3.79, and 4.55 eV, respectively. It suggests that electrons are transferred from CoP to Ni_2_P in CoP@Ni_2_P heterojunction whereas electrons are transferred from Fe‐Ni_2_P to Fe‐CoP in Fe‐CoP@Fe‐Ni_2_P heterojunction, which is consistent with XPS observations.

According to the d‐band center (ε_d_) theory, anti‐bonding states are generated from the coupling between d‐orbitals of the metal atom and 1s‐orbitals of H atom (HER) or 2p‐orbitals of O atom (OER).^[^ ^53^, ^54^ ^]^ The closer ε_d_ of metal site to the Fermi level the lesser electrons in the anti‐bonding state, thus stronger adsorption of the intermediates onto the metal site.^[^ ^55^ ^]^ As the difference between ε_d_ and Fermi level positively correlates with the band gap,^[^ ^56^, ^57^ ^]^ we determined the bandgaps of the catalysts using UV–vis diffuse reflection (Figure S13, Supporting Information). The bandgap of CoP@Ni_2_P is wider than pure CoP but narrower than pure Ni_2_P. It suggests that the adsorption strength of hydrogen intermediate follows the order of CoP > CoP@Ni_2_P > Ni_2_P. Sabatier principle,^[^ ^58^ ^]^ which posits that the adsorption of intermediates on the active site should neither be too strong nor too weak, explains the superior HER performance of CoP@Ni_2_P. Similarly, Fe‐CoP@Fe‐Ni_2_P, which possesses a moderate band gap compared to pure Fe‐CoP and Fe‐Ni_2_P, gives the best OER performance.

Combining the results of UPS and UV–vis, we draw the energy band diagrams of CoP, Ni_2_P (Figure 5b) and Fe‐CoP, Fe‐Ni_2_P (Figure 5c). The CoP@Ni_2_P is a typical Type‐I heterojucntion, which is infavorable for separation of electrons and holes.^[^ ^59^ ^]^ The ΔΦ between CoP and Ni_2_P is 0.18 eV, providing a weak driving force for electron transfer from CoP to Ni_2_P. Taken together, the CoP@Ni_2_P heterojucntion structure is not conducive for the formation of distinct nucleophilic and electrophilic sites.^[^ ^18^ ^]^ In contrast, Fe doping transforms a type‐I heterojunction (CoP@Ni_2_P) to a type‐II heterojunction (Fe‐CoP@Fe‐Ni_2_P) which is favorable for separation of electrons and holes.^[^ ^59^ ^]^ In addition, the ΔΦ between Fe‐CoP and Fe‐Ni_2_P is much larger (0.76 eV), signifying that Fe doping not only alters the direction of the interfacial electric field but also increases the energy level difference between the heterogeneous phosphides, thereby effectively accelerating the electron transfer from Fe‐Ni_2_P to Fe‐CoP, resulting in the electrophilic Ni sites for OER.

We then constructed the DFT models of CoP@Ni_2_P heterojunction as the HER cathode and Fe‐CoP@Fe‐Ni_2_P/NF, Fe‐CoOOH@Fe‐NiOOH heterojunctions (Fe‐CoP@Fe‐Ni_2_P/NF undergoes in situ oxidation as shown in Figure S10–S12, Supporting Information) as the OER cathode (Figure 5d). For the Fe‐doped heterojunction, considering OER commonly takes place on the catalyst surface and XPS is a surface analysis technique,^[^ ^60^, ^61^ ^]^ we assumed a molar ratio of Co:Fe:Ni = 1:1:1 because the atomic ratio of Co:Fe:Ni measured by XPS is 4.53:5.46:4.73, and that Fe is equally doped into both CoP and Ni_2_P (or CoOOH and NiOOH) (Figures S3b and S4, Supporting Information). The charge density difference analysis (Figure 5d) reveals a clear electron transfer from CoP to Ni_2_P, leading to electron enrichment at the Ni sites which is favorable for adsorption of H intermediate (H*). With Fe doping, a reverse electron transfer occurs on Fe‐CoP@Fe‐Ni_2_P, resulting in electron deficiency around Ni sites. During OER, Fe‐CoP@Fe‐Ni_2_P was oxidized to Fe‐CoOOH@Fe‐NiOOH. However, the direction of the interfacial electric field remains unchanged, indicating that the surface reconstruction does not adversely impact the electron transfer at the interface. As shown in Figure 5e, the calculated density of state (DOS) shows that ε_d_ of Fe‐CoP@Fe‐Ni_2_P upshifts toward Fermi level compared to CoP@Ni_2_P, leading to favorable adsorption of O intermediate (*OH).^[^ ^62^ ^]^ Furthermore, the DOS of Fe‐CoOOH@Fe‐NiOOH exhibits a further upshift, suggesting that surface reconstruction enhances the adsorption of O intermediate (*OH). Thus, Fe‐CoOOH@Fe‐NiOOH is desirable for OER whereas CoP@Ni_2_P is suitable for HER.

The zero Gibbs free energy of H* adsorption (ΔG_H*_) is ideal for HER because of the optimal balance between adsorption and desorption processes.^[^ ^63^ ^]^ For Ni site in the CoP@Ni_2_P heterojunction, ΔG_H*_ is calculated to be 0.048 eV (Figure 5f), which is closer to 0 eV than that for Co and P sites in the heterojunction, Co and P sites in pure CoP, and Ni and P sites in pure Ni_2_P (Figures S14 and S15, Supporting Information). After Fe doping, both Co site in CoP and Ni site in Ni_2_P experience an increase in ΔG_H*_, suggesting that Fe doping compromises the HER kinetics (Figure S15, Supporting Information). The interfacial electric field in Fe‐CoOOH@Fe‐NiOOH is reversed as compared to that of CoP@Ni_2_P, making Ni site electron‐deficient thus an electrophilic site for adsorbing O intermediates (*OH, *O, and *OOH) during OER. Compared among all the metal sites in Fe‐CoOOH@Fe‐NiOOH, Fe‐CoOOH, Fe‐NiOOH, CoOOH, NiOOH (Figures S16 and S17, Supporting Information), Ni site in Fe‐CoOOH@Fe‐NiOOH exhibits lowest ΔG in each reaction step during OER, especially for the RDS (the conversion of O* to OOH*), indicating that Fe‐CoOOH@Fe‐NiOOH gives the fastest OER kinetics with Ni as the active site (Figure 5g). Taken together, Figure 5h illustrates the catalytic mechanism for the water splitting.

### 2.6 Overall Water Splitting Performance

**Figure 6:**
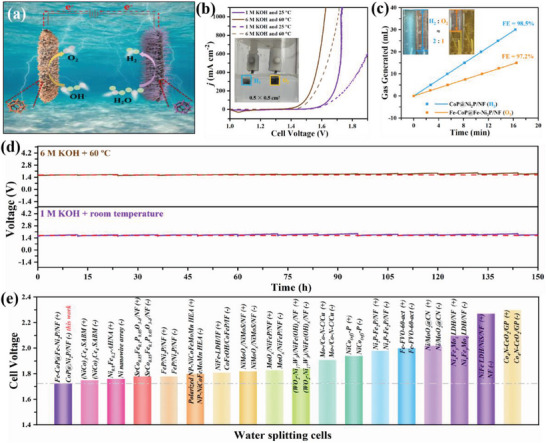
Electrocatalytic activities of samples for overall water splitting: a) Scheme of Fe‐CoP@Fe‐Ni_2_P/NF (+) ll CoP@Ni_2_P/NF (‐) water splitting cell. b) Reverse polarization curves of cells in 1 m KOH at 25 °C and in 6 m KOH at 60 °C (solid line: Fe‐CoP@Fe‐Ni_2_P/NF (+) ll CoP@Ni_2_P/NF (‐), dotted line: RuO_2_ (+) ll Pt/C (‐)). c) Generated H_2_ (blue line) and O_2_ (orange line) over time. Insert shows that the generated H_2_ and O_2_ are 30 and 15 mL, respectively. d) Chronopotentiometric measurement for 150 h at the constant *j*_1000_ in 1 m KOH, 25 °C and 6 m KOH, 60 °C (Replace the electrolyte regularly, with a interval of every 12 h). e) Comparison of the cell voltage with the reported electrocatalysts in 1 m KOH.

We constructed a water splitting cell with CoP@Ni_2_P/NF as the cathode and Fe‐CoP@Fe‐Ni_2_P/NF as the anode (**Figure** **6**a). This cell only requires a voltage of 1.724 V to drive *j*_1000_ in the experimental condition (1 m KOH and room temperature) (Figure 6b), and it can be further reduced to 1.620 V in the industrial water splitting condition (6 m KOH and 60 °C). The performance is superior to that of the commercial RuO_2_ (+) ll Pt/C (‐) cell in both conditions, demonstrating its potential for practical use. The Faraday efficiencies calculated from measured H_2_ and O_2_ production (Figure 6c) are 98.5% and 97.2%, respectively, with a molar ratio close to 2:1, which is comparable to the recently reported self‐supported electrocatalyst (Table S5, Supporting Information). Additionally, the cell remained stable after 150 h of operation at *j*_1000_ (Figure 6d) in both experimental and industrial conditions. At both experimental and industrial conditions, our cell is comparable or outperforms the state‐of‐the‐art water splitting cells equipped with self‐supporting electrodes in terms of both performance and stability (Figure 6e; Tables S6 and S7, Supporting Information). The cell voltage of Fe‐CoP@Fe‐Ni_2_P/NF (+) ll CoP@Ni_2_P/NF (‐) at 400 mA cm^−2^ is 1.68 V at industrial condition (without iR compensation, Figure S18, Supporting Information) and the FE of H_2_ production is 98.5%, indicating an energy consumption is ≈4.1 kWh m^−3^ H_2_, which is lower than the cell equiped by commercial Raney Ni electrode (2.05 V to reach 400 mA cm^−2^, corresponding energy consumption is 4.9 kWh m^−3^ H_2_) and the target set by the US Department of Energy.^[^ ^64^, ^65^ ^]^

## 3 Conclusion

In summary, self‐supported HER cathode (CoP@Ni_2_P/NF) and OER anode (Fe‐CoP@Fe‐Ni_2_P/NF) were readily synthesized. Comprehensive experimental and theoretical investigations demonstrate that 1) an interfacial electric field is established between CoP@Ni_2_P heterojunction because of the difference in work functions of the two phosphides (∆Φ), rendering in the electron‐rich Ni sites favorable for H* adsorption in HER; 2) interfacial engineering by Fe doping reverses and enlarges the interfacial electric field thereby turning the Ni sites into electron‐deficient, favoring O intermediates adsorption during OER. The overall water splitting cell equipped with the two electrodes only required a low voltage of 1.724 V to drive *j*_1000_ with long‐term stability, showing the potential for industrial applications. This study demonstrates that doping metallic element is an effective strategy to modulate interfacial electric field of a heterojunction catalyst whereby altering its catalytic properties.

## 4 Experimental Section

### Materials

Iron(ІІІ) nitrate nonahydrate (Fe(NO_3_)_3_·9H_2_O) and urea were purchased from Xilong Scientific. Cobalt(II) nitrate hexahydrate (Co(NO_3_)_2_·6H_2_O) was obtained from Adamas Reagent. Potassium hydroxide (KOH, ≥90.0%) and sodium hypophosphite hydrate (NaH_2_PO_2_·H_2_O) were received from Shanghai Titanchem. Acetone, hydrochloric acid (HCl) and ethanol were purchased from Shanghai Lingfeng Chemical. Nafion solution (5 wt.%), RuO_2_ and Pt/C were purchased from Sigma–Aldrich.

### Synthesis

*Ni_2_P/NF*: Nickle foam (NF, 3 × 1 cm^2^) was washed by 3 m HCl, acetone and ethanol for several times, then dried at 60 °C under vacuum overnight. The cleaned NF was then placed inside a tube furnace with 6 mmol of NaH_2_PO_2_·H_2_O being placed upstream. The tube furnace was heated to 300 °C with a rate of 2 °C min^−1^ and kept for 2 h under argon atmosphere to produce phosphatized NF (Ni_2_P/NF).

*Fe‐Ni_2_P/NF*: Cleaned NF (3×4 cm^2^), 1 mmol of Fe(NO_3_)_3_·9H_2_O and 25 mmol of urea were put in 50 mL of deionized water under ultrasonication for 30 min, then transferred into 100 mL Teflon‐lined autoclave for 12 h heating at 90 °C. The obtained Fe‐doped nickel carbonate hydroxide (Fe‐Ni‐CH/NF) was washed by deionized water and ethanol for several times and dried at 60 °C under vacuum overnight. Subsequently, Fe‐Ni‐CH/NF (3 × 1 cm^2^) was phosphatized to produce Fe‐Ni_2_P/NF, the phosphorization process was carried out in the same way as for Ni_2_P/NF.

*CoP@Ni_2_P/NF and Fe‐CoP@Fe‐Ni_2_P/NF*: Cleaned NF (3 × 4 cm^2^) was used as support to prepared phosphide heterojunctions, and the hydrothermal and phosphating methods were same as for Fe‐Ni_2_P/NF, but with different metal salt precursors (CoP@Ni_2_P/NF: 5 mmol of Co(NO_3_)_2_·6H_2_O and Fe‐CoP@Fe‐Ni_2_P/NF: 1 mmol of Fe(NO_3_)_3_·9H_2_O and 4 mmol of Co(NO_3_)_2_·6H_2_O). Excess Fe‐doped heterojunction samples were prepared by increasing the molar ratio of Fe/Co precursor (5/3 mmol of Fe(NO_3_)_3_·9H_2_O and 10/3 mmol of Co(NO_3_)_2_·6H_2_O; 5/2 mmol of Fe(NO_3_)_3_·9H_2_O and 5/2 mmol of Co(NO_3_)_2_·6H_2_O).

*Mechanically mixed samples (CoP+Ni_2_P/NF and Fe‐CoP+Fe‐Ni_2_P/NF)*: CoP or Fe‐CoP powder was synthesized using the same hydrothermal and phosphating method as for CoP@Ni_2_P/NF or Fe‐CoP@Fe‐Ni_2_P/NF (the molar ratio of Fe/Co precursor is 1 mmol/4 mmol), but without the addition of NF. Specifically, 0.1 g of hydrothermal product (Co‐CH or Fe‐Co‐CH) was used for phosphorization, the process was carried out in the same way as for Ni_2_P/NF. 5 mg of CoP or Fe‐CoP powders were dispersed in a mixed solution (20 µL of Nafion, 490 µL of deionized water and 490 µL of ethanol) under ultrasonication for 30 min to form catalyst inks. The CoP or Fe‐CoP inks were then coated on the surface of Ni_2_P/NF or Fe‐Ni_2_P/NF (1 × 1 cm^2^) and were dried at 60 °C under vacuum overnight to produce mechanically mixed samples (CoP+Ni_2_P/NF and Fe‐CoP+Fe‐Ni_2_P/NF).

*Pt/C/NF and RuO_2_/NF*: The preparation procedures for Pt/C/NF and RuO_2_/NF were the same as for CoP+Ni_2_P/NF, except that the inks were prepared with 5 mg of Pt/C or RuO_2_ powders and the substrate used was a clean NF (1 × 1 cm^2^).

### Characterization

X‐ray diffraction (XRD) patterns were obtained using a SmartLab diffractometer (Smartlab3KW, Rigaku) with a scanning range of 5° to 80° (2θ) and a scanning rate of 10°/min. The structure and morphology of the as‐prepared samples were characterized using scanning electron microscopy (SEM, JSM7800F, JEOL), focus ion beam‐scanning electron microscope (FIB‐SEM, FEI Scios 2 HiVac), high‐resolution transmission electron microscopy (HRTEM, JEM2100F, JEOL). The elemental states of the as‐prepared samples were analyzed using X‐ray photoelectron spectroscopy (XPS, Thermo Scientific, USA). Raman spectrum were measured on a Renishaw inVia Reflex Raman spectrometer (under an excitation of 785 nm laser). The Ultraviolet photoelectron spectroscopy (UPS) was conducted using a ThermoFisher ESCALAB 250Xi instrument equipped with an ultraviolet photoelectron spectrometer (HeI, 21.22 eV). Additionally, UV–vis diffuse reflectance spectra (UV–vis) were obtained using a UV‐2600 instrument. All samples were loaded onto NF for UPS and UV–vis tests.

### Electrochemical Measurements

All electrochemical performance tests were carried out using a CHI 660D electrochemical workstation (CH Instruments, China) at room temperature (or otherwise specified temperature). For HER and OER tests, a three‐electrode system was used, with the as‐prepared samples (working area, 0.5 × 0.5 cm^2^) as the working electrode, Ag/AgCl (3.5 m KCl, 0.2046 V vs normal hydrogen electrode) as the reference electrode, and graphite as the counter electrode. For overall water splitting, a two‐electrode system was used, with CoP@Ni_2_P/NF as the cathode and Fe‐CoP@Fe‐Ni_2_P/NF as the anode (showing in the inserted image in Figure 6b). Specifically, the working electrode clip was connected to the anode, whereas the counter and reference electrode clips were simultaneously connected to the cathode. All systems were tested in 1 m KOH (pH = 13.6), unless otherwise specified. The potential versus reversible hydrogen electrode (RHE) was calculated from the measured potentials using Equation (9):^[^ ^66^ ^]^

(9) $$E\left(\mathrm{vs}\;RHE\right)\;=\;E\left(\mathrm{vs}\;\mathrm{Ag}/\mathrm{AgCl}\right)\;+\;0.2046\;+\;0.059\;\times \;\mathrm{pH}$$

Particularly, the overpotential (η) for OER was converted via Equation (10):^[^ ^66^ ^]^

(10) $$\eta \;=\;E\left(\mathrm{vs}\;RHE\right)\;-1.23$$

The electrocatalytic activities were evaluated using polarization curves based on linear sweep voltammetry (LSV) with a scan rate of 0.5 mV s^−1^. For OER and overall water splitting, reverse polarization curves were used to determine the overpotential, which avoids overestimation of the catalytic activity caused by the strong oxidation peak originating from metal species in the polarization curves. Tafel slopes were calculated from iR‐corrected polarization curves via Equation (11):^[^ ^66^ ^]^

(11) $$\eta \;=\;b\;\log\left|j\right|\;+\;a$$

where η is overpotential, b is tafel slope, *j* is current density.

The double‐layer capacitances (C_dl_) were calculated based on cyclic voltammetry (CV) in non‐faradaic region, with different scan rates of 2, 4, 6, 8, and 10 mV s^−1^. The C_dl_ could be calculated via Equation (12):^[^ ^67^ ^]^

(12) $$C_{\mathrm{dl}}=\;\left|j_{a}-j_{b}\right|/\left(2\times v\right)\;=\;\Delta j/\left(2\times v\right)$$

where *j*_a_ and *j*_b_ are the two current density recorded at the middle of the potential range of CV curves, and *v* is the scan rate.

Electrochemical impedance spectroscopy (EIS) was measured at potential (without conversion via RHE) of −1.2 V for HER, 0.52 V for OER and 1.6 V for overall water splitting. The amplitude was set to 5 mV and the frequency range was from 0.1 Hz to 1 × 10^5^ Hz.

The Faradaic efficiency (FE) of the cathode and anode in the water splitting cell was calculated using the drainage method at 20 °C. The water splitting process was conducted in an H‐type electrolytic cell, with a proton exchange membrane between the cathode and anode to prevent diffusion of H_2_ and O_2_ to the opposite electrode, and the test current was set as 0.25 A. The generated H_2_ and O_2_ were collected in cylinders which were filled with methylene blue and methyl orange solutions, respectively, to mark cylinder graduations. The time taken (t) for the release of 5 mL of H_2_ or 2.5 mL of O_2_ was recorded, and FE was calculated using the following equation:^[^ ^68^ ^]^

(13) $$FE\;=\;\frac{znF}{It}$$

where z represents the number of electron transfer (H_2_, z = 2; O_2_, z = 4), n is the gas generated amount (mol), F is Faraday constant (96 485 C mol^−1^), I is test current (A).

The electrochemical stabilities were evaluated through chronopotentiometric measurements. All polarization and chronopotentiometric curves have subjected to iR‐compensation to eliminate the influence of ohmic loss, unless otherwise specified.^[^ ^67^ ^]^ Ohmic loss arises from various sources, including wiring, substrate, catalyst material, and solution resistances. Collectively, these resistances form the series resistance (R_s_), which is determined by fitting the EIS Nyquist plot before conducting LSV and chronopotentiometric tests. The corrected potential (E_corrected_) can be obtained by the equation E_corrected_ = E_initial_‐i×R_s_, where i is the current. As the Tafel slope is directly linked to the overpotential,^[^ ^69^ ^]^ such 100% iR compensation is needed to accurately determine the Tafel slope. All the R_s_ are listed in Table S8 (Supporting Information) and the original LSV curves are plotted in Figure S18 (Supporting Information).

Since gas collection was conducted under the approximately standard condition, the actual energy consumption (E_con_, kWh m^−3^ H_2_) was calculated at standard condition (STP) using the following equation:^[^ ^70^ ^]^

(14) $$E_{\mathrm{con}}\;=\;2F\times \frac{1}{3.6\cdot 10^{6}}\times E_{app}\times \frac{P}{RT}\times \frac{1}{FE}$$

where 2 is the number of electron transfer per H_2_, F is Faraday constant (96 485 C mol^−1^), 3.6 × 10^−6^ represents a factor to convert Joules to kWh, E_app_ is the cell voltage, P is the pressure (101 325 Pa), R is the ideal gas law constant (8.314 J mol^−1^ K^−1^), T is the temperature (273.15 K), FE is the Faradaic efficiency of H_2_ production.

## Conflict of Interest

The authors declare no conflict interest.

## Author Contributions

Z.L. conceived the project, designed, and performed experiments, analyzed the data, prepared figures, and wrote the manuscript. C.X. assisted in experiments and characterizations. Z.Z. conceived the project and designed experiments. S.X. and L.L. performed the theoretical calculation. D. L. assisted in characterizations. P.C., X. M., and X.D. provided supervision, writing‐reviewing, and editing.

## Supporting information

Supporting Information

## Data Availability

The data that support the findings of this study are available from the corresponding author upon reasonable request.
