# Supplementary material for: Reversing the Interfacial Electric Field in Metal Phosphide Heterojunction by Fe‐Doping for Large‐Current Oxygen Evolution Reaction
Source: Adv Sci (Weinh). 2024 Apr 8;11(21):2308477. doi: 10.1002/advs.202308477 (PMC11151033; doi:10.1002/advs.202308477)
Supplement: Supplementary file 1 — Supporting Information [file ADVS-11-2308477-s001.pdf]

ADVANCED SCIENCE
Open Access

Supporting Information

for *Adv. Sci.*, DOI 10.1002/advs.202308477

Reversing the Interfacial Electric Field in Metal Phosphide Heterojunction by Fe-Doping for Large-Current Oxygen Evolution Reaction

Zhong Li, Chengshuang Xu, Zheye Zhang, Shan Xia, Dongsheng Li, Liren Liu, Peng Chen* and Xiaochen Dong**

Supporting Information

Reversing the Interfacial Electric Field in Metal Phosphide Heterojunction by Fe-doping for Large-Current Oxygen Evolution Reaction

Zhong Li, Chengshuang Xu, Zheye Zhang, Shan Xia, Dongsheng Li, Liren Liu, Peng Chen*, Xiaochen Dong**

Z. Li

Center for Rehabilitation Medicine, Rehabilitation & Sports Medicine Research Institute of Zhejiang Province, Department of Rehabilitation Medicine, Cancer Center, Zhejiang Provincial People's Hospital (Affiliated People's Hospital), Hangzhou Medical College, Hangzhou, Zhejiang 310014, China.

Z. Li, C. Xu, S. Xia, L. Liu, X. Dong

Key Laboratory of Flexible Electronics (KLOFE) & Institute of Advanced Materials (IAM), School of Flexible Electronics (Future Technologies), School of Physical and Mathematical Sciences, Nanjing Tech University (NanjingTech), Nanjing 211816, China.

E-mail: lrliu@njtech.edu.cn; iamxcdong@njtech.edu.cn

Z. Zhang, P. Chen

School of Chemistry, Chemical Engineering and Biotechnology, Institute for Digital Molecular Analytics and Science, Nanyang Technological University, 637457, Singapore.

E-mail: chenpeng@ntu.edu.sg

D. Li

College of Materials and Chemical Engineering, Key Laboratory of Inorganic Nonmetallic Crystalline and Energy Conversion Materials, China Three Gorges University, Yichang 443002, China

First-principles calculations. The calculations were performed within the density functional theory framework with the Perdew-Burke-Ernzerhof functional.[1] A vacuum space of 20 Å was set to eliminate spurious interaction between two surfaces in adjacent periodic images. The core electron-ion interaction was described by the projector augmented wave potential,[1,2] and the cut-off energy of the plane-wave basis was set to be 420 eV. The Brillouin zone integration was sampled using 7×7×1 k points for the structural optimization, 3×3×1 k points for AIMD simulations and 16 k points for electronic calculations. The atomic positions were optimized until the maximum force on each atom was less than 0.01 eV $Å^{-1}$.

Theoretical calculation of ΔG. Theoretical calculation of ΔG. The calculations of ΔG of HER/OER reactions were also performed by using the Perdew-Burke-Ernzerhof generalized gradient approximation method. A kinetic energy cut-off was set to 500 eV for the plane-wave expansion in this set of calculations. For the HER reactions, the Gibbs free energies ΔG at 300 K were calculated as $\Delta G = \Delta E + \Delta E_{ZPE} - T\Delta S$, where ΔE is the adsorption energy of the hydrogen atom on the system with respect to the gas phase, T is the temperature, ΔS is the entropy of a H atom adsorbed on the substrate. The harmonic approximation was employed for the vibrational analysis of adsorbates, while gas phase reference molecules were treated as ideal. For the OER reaction pathways, the *O, *OH and *OOH absorbed structures on the substrate were also optimized by the the Perdew-Burke-Ernzerhof method.

Reference

[1] J. P. Perdew, K. Burke, M. Ernzerhof, *Phys. Rev. Lett.* **1996**, *77*, 3865-3868.

[2] G. Kresse, D. Joubert, *Phys. Rev. B* **1999**, *59*, 1758-1775.

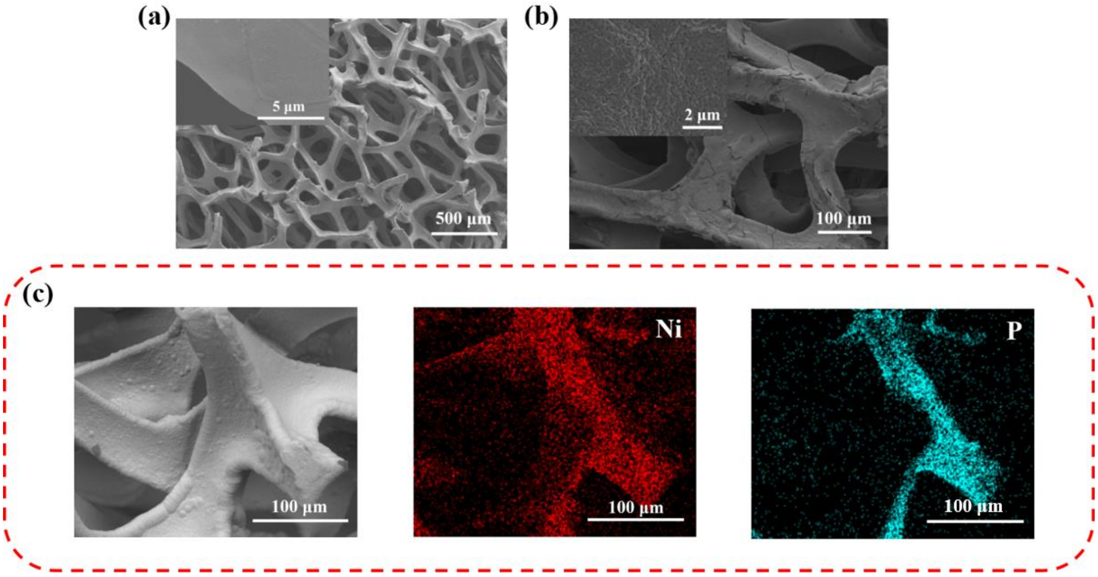

Figure S1. (a) SEM image of NF (inserted image is enlarged sample). (b) SEM image of Ni_2P/NF (inserted image is enlarged sample). (c) Energy Dispersive X-Ray Spectroscopy (EDX) mapping of Ni_2P/NF.

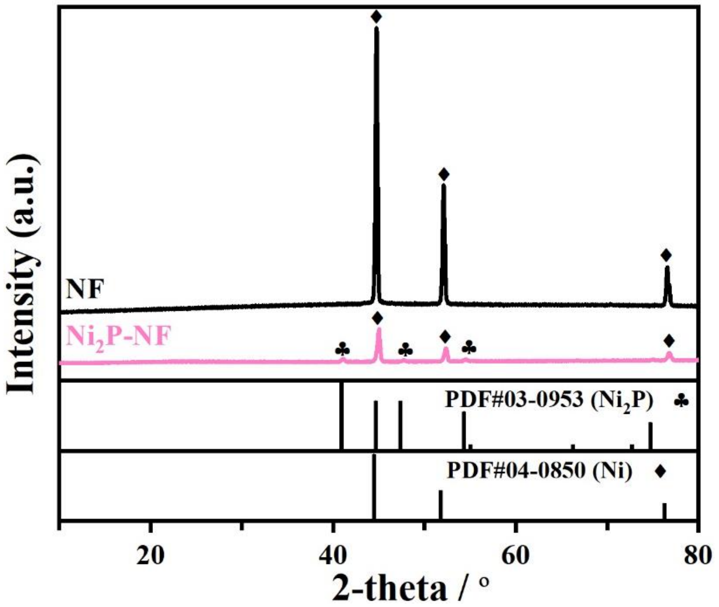

Figure S2. XRD patterns of NF and Ni_2P/NF.

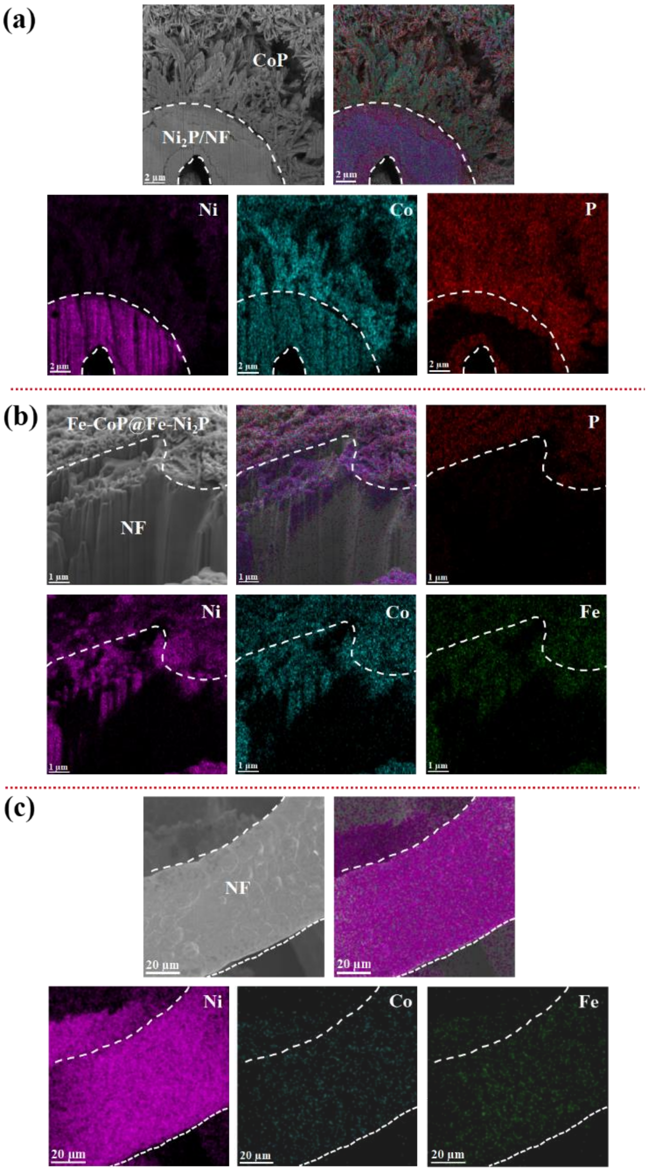

Figure S3. Focus ion beam-scanning electron microscope (FIB-SEM) mappings of (a) CoP@Ni_2P/NF cross section. (b) Fe-CoP@Fe-Ni_2P/NF cross section. (c) EDX mapping of NF. (Notably, few Co and Fe impurities can be found in pure NF. However, no discernible peaks corresponding to metallic Co and Fe are detected in XRD pattern of NF (Figure S2), as well as in CoP@Ni_2P/NF (Figure 1e) and Fe-CoP@Fe-Ni_2P/NF (Figure 1j). Furthermore, there are no indications of metallic Co and Fe in the XPS spectrum of CoP@Ni_2P/NF and Fe-CoP@Fe-Ni_2P/NF (Figure 4a,c). This suggests that the few Co and Fe impurities does not impede the formation of heterojunction catalysts on NF surface.）

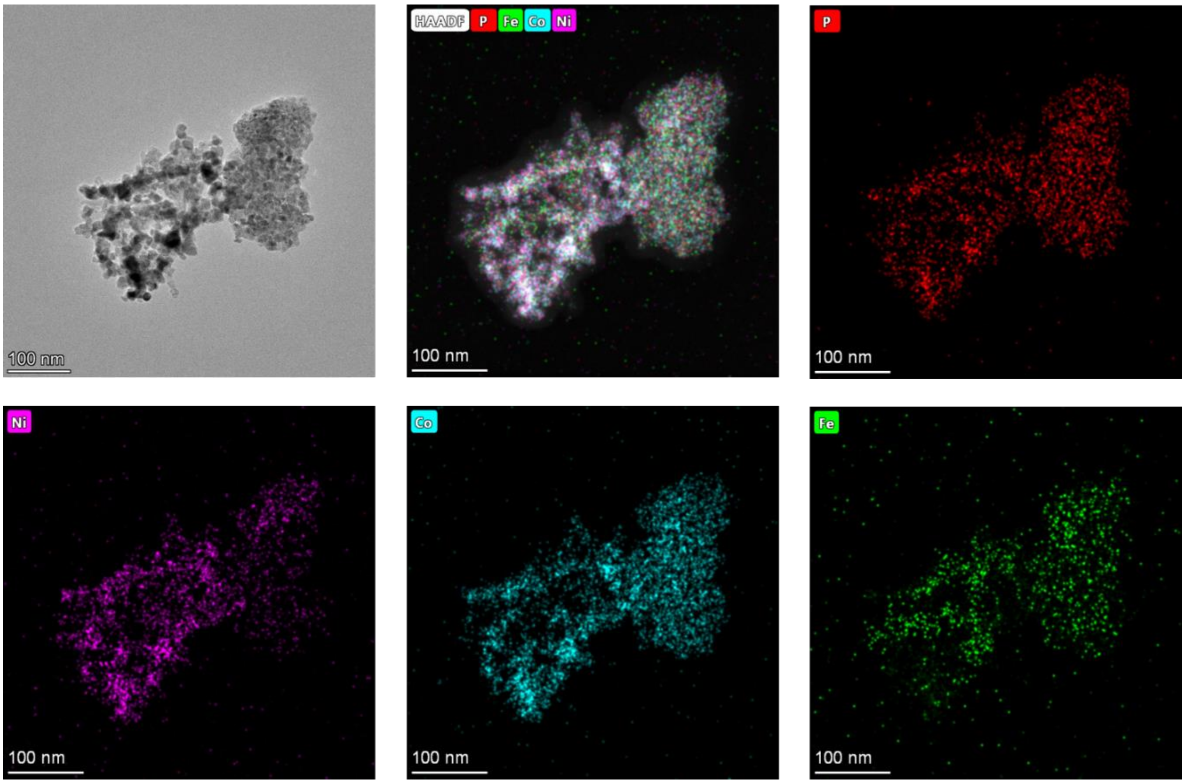

Figure S4. TEM mapping of Fe-CoP@Fe-Ni_2P powder peeled off from NF by ultrasound treatment.

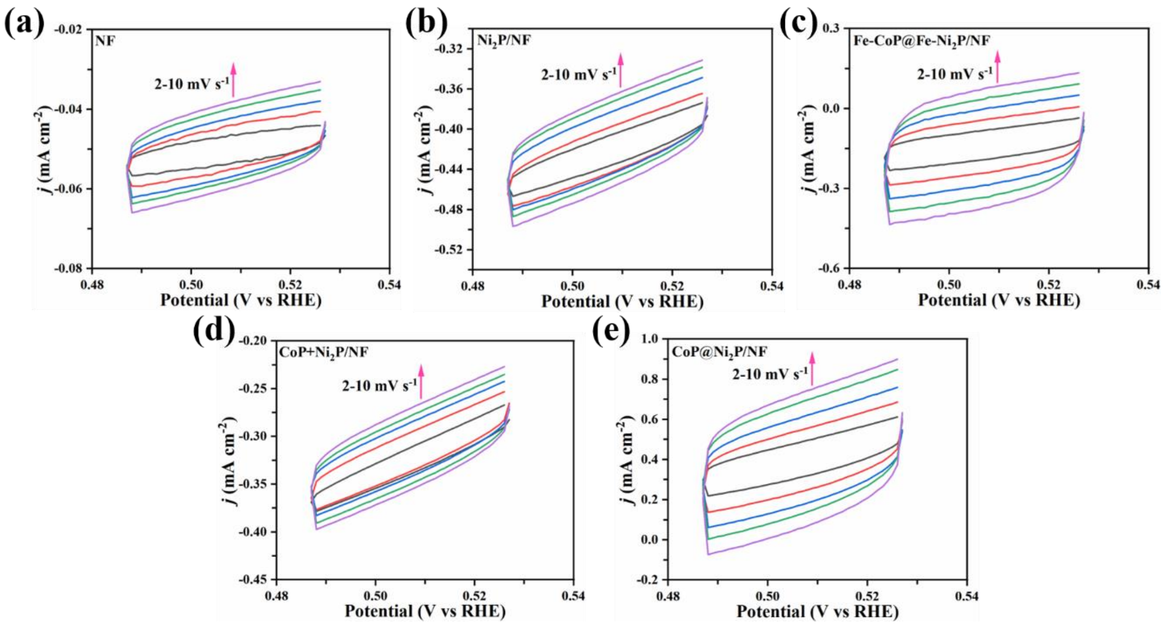

Figure S5. (a - e) Cyclic voltammetry curves of catalysts with different scan rates (2, 4, 6, 8, 10 mV s^{-1}) in the nonfaradaic region for HER.

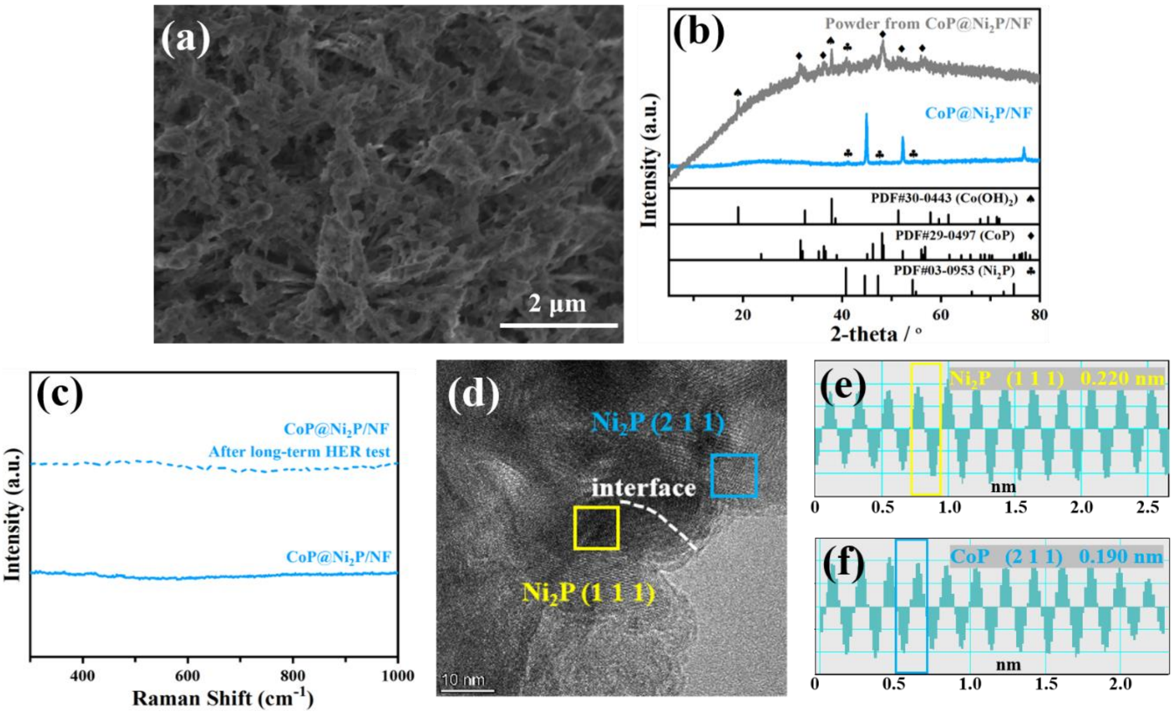

Figure S6. (a) SEM image of CoP@Ni_2P/NF after long-term HER test. (b) XRD patterns of CoP@Ni_2P/NF after long-term HER test. (c) Raman spectra of CoP@Ni_2P/NF before and after long-term HER test. (d) HRTEM image of CoP@Ni_2P/NF after long-term HER test (dash line indicates the interface between Ni_2P and CoP). (e) and (f) Corresponding line scan of inverse fast Fourier transform (FFT) of facets of Ni_2P and CoP.

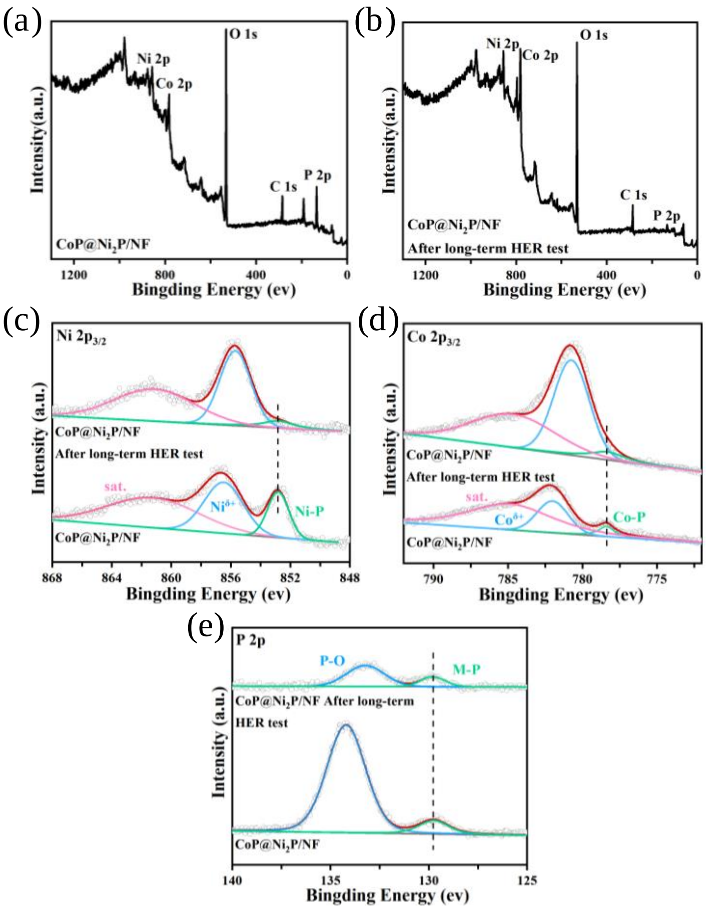

Figure S7. (a) The XPS survey spectrum of CoP@Ni_2P/NF. (b) The XPS survey spectrum of CoP@Ni_2P/NF after long-term HER test. (c) Ni $2p_{3/2}$ XPS spectra of CoP@Ni_2P/NF before and after long-term HER test. (d) Co $2p_{3/2}$ XPS spectra of CoP@Ni_2P/NF before and after long-term HER test. (e) P 2p XPS spectra of CoP@Ni_2P/NF before and after long-term HER test.

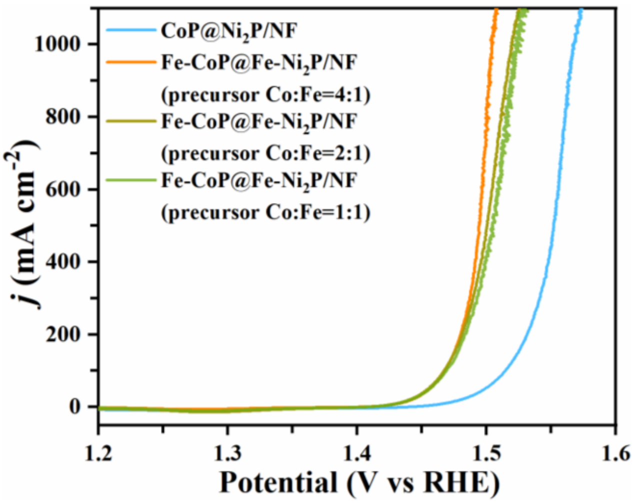

Figure S8. The OER polarization curves of CoP@Ni_2P/NF and Fe-CoP@Fe-Ni_2P/NF prepared by precursors with different Co/Fe molar ratios.

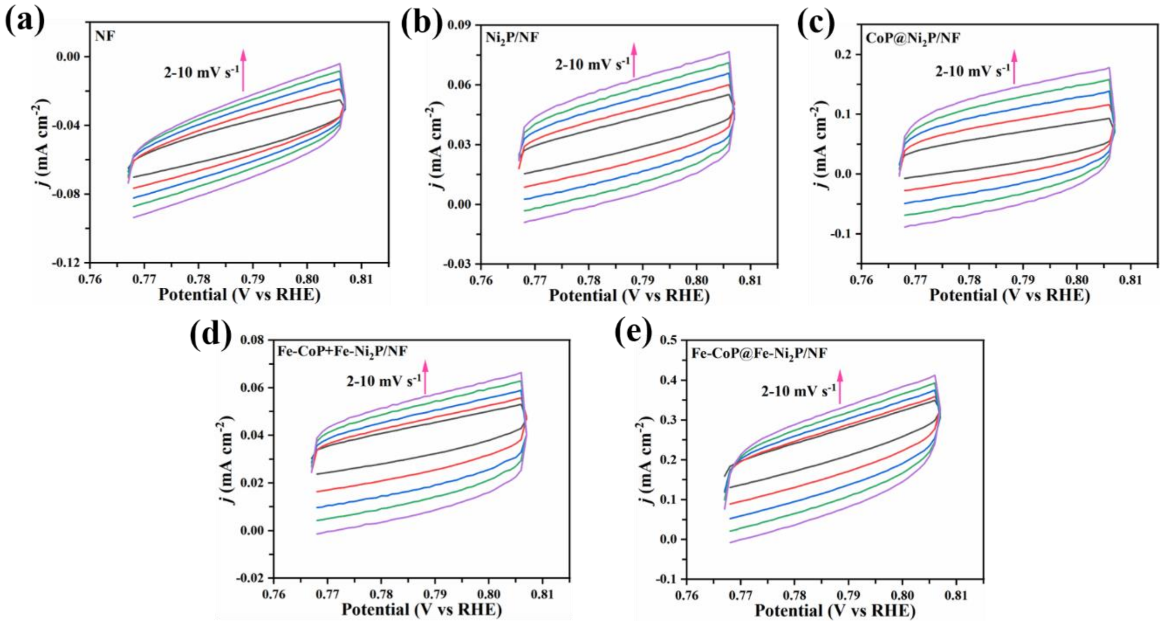

Figure S9. (a - e) Cyclic voltammetry curves of catalysts with different scan rates (2, 4, 6, 8, 10 mV s^{-1}) in the nonfaradaic region for OER.

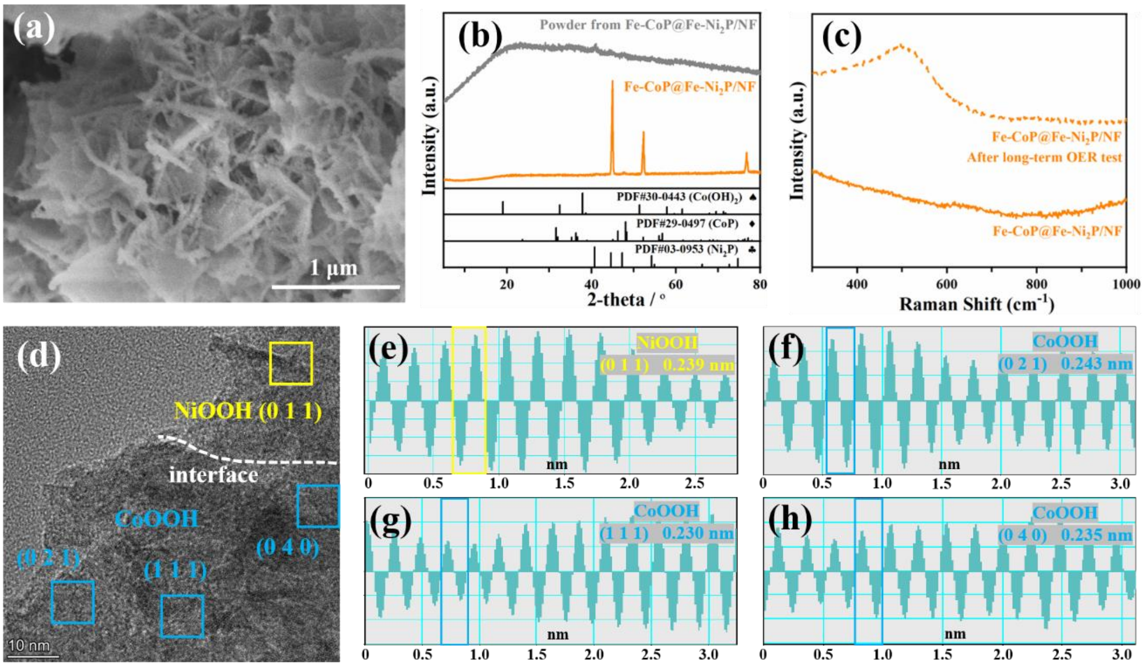

Figure S10. (a) SEM image of Fe-CoP@Fe-Ni_2P/NF after long-term OER test. (b) XRD patterns of Fe-CoP@Fe-Ni_2P/NF after long-term OER test. (c) Raman spectra of Fe-CoP@Fe-Ni_2P/NF before and after long-term OER test. (d) HRTEM image of Fe-CoP@Fe-Ni_2P/NF after long-term OER test (the Fe-CoP@Fe-Ni_2P was in situ transformed into Fe-CoOOH@Fe-NiOOH during OER, dash line indicates the interface between Fe-CoOOH@Fe-NiOOH). (e) Corresponding line scan of inverse fast Fourier transform (FFT) of facet of NiOOH (Refer to PDF#00-027-0956). (f)-(h) Corresponding line scan of

inverse fast Fourier transform (FFT) of facets of CoOOH (Refer to PDF#00-026-0480).

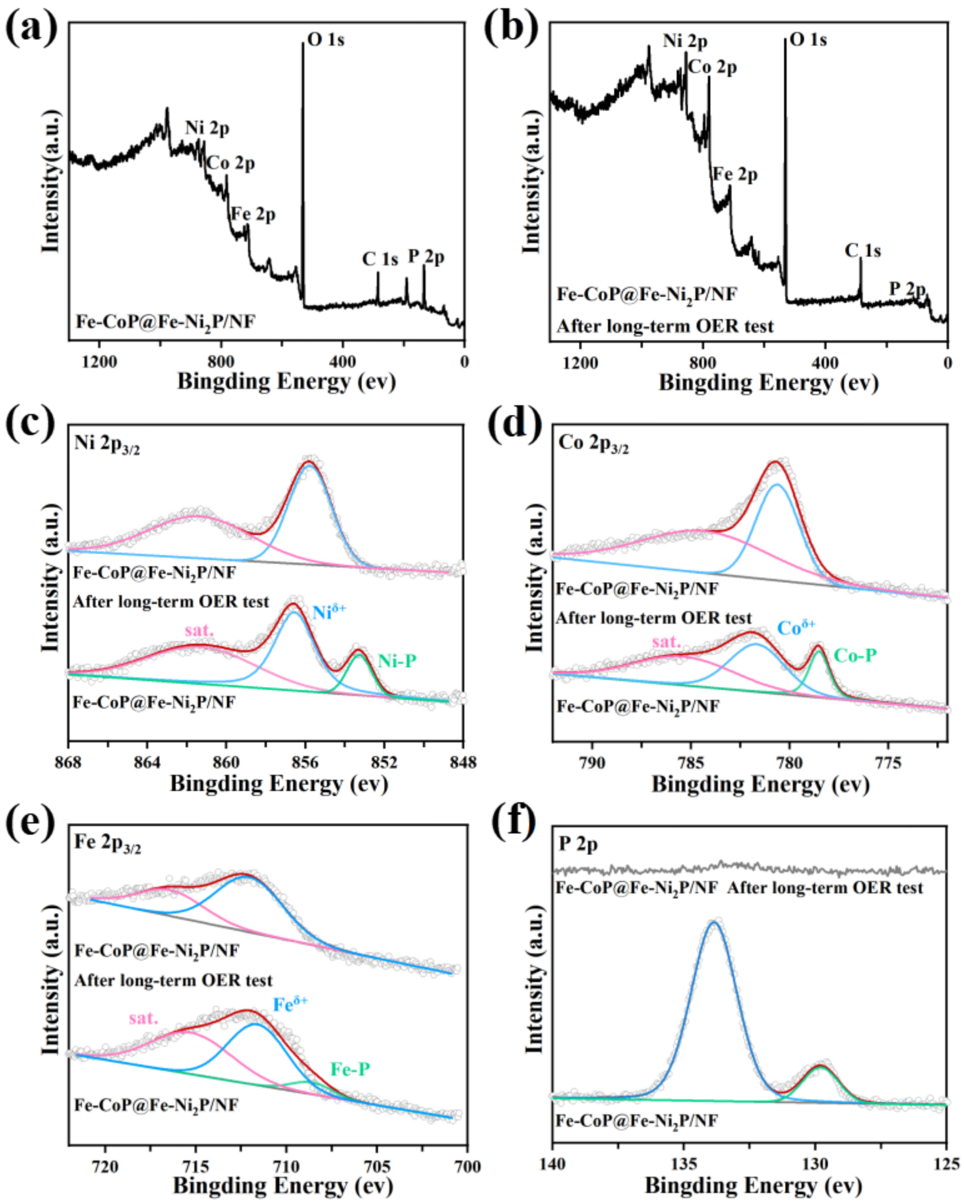

Figure S11. (a) The XPS survey spectrum of Fe-CoP@Fe-Ni_2P/NF. (b) The XPS survey spectrum of Fe-CoP@Fe-Ni_2P/NF after long-term OER test. (c) Ni $2p_{3/2}$ XPS spectra of Fe-CoP@Fe-Ni_2P/NF before and after long-term OER test. (d) Co $2p_{3/2}$ XPS spectra of Fe-CoP@Fe-Ni_2P/NF before and after long-term OER test. (e) Fe $2p_{3/2}$ XPS spectra of Fe-CoP@Fe-Ni_2P/NF before and after long-term OER test. (e) P 2p XPS spectra of Fe-CoP@Fe-Ni_2P/NF before and after long-term OER test.

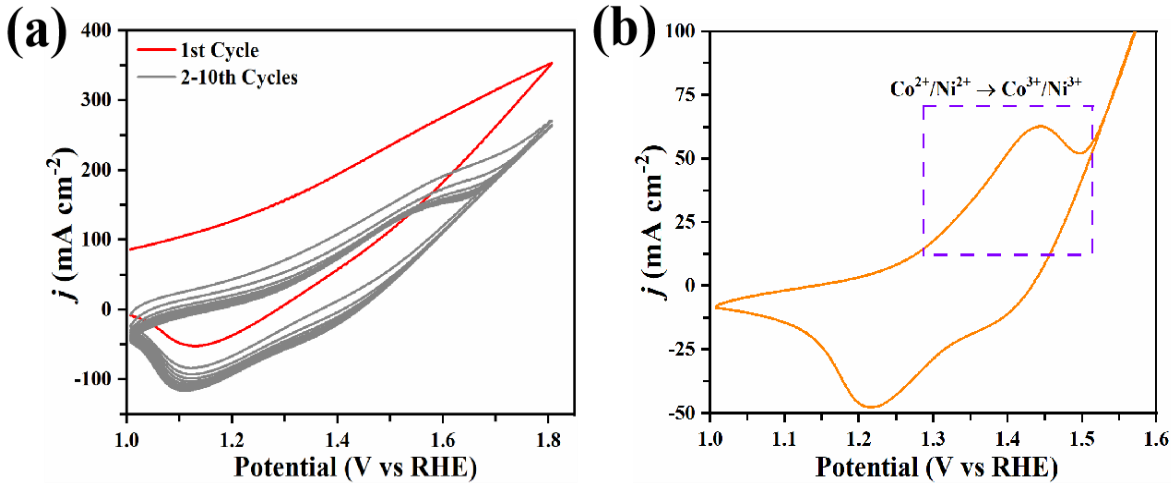

Figure S12. (a) The CV curves for activation of fresh Fe-CoP@Fe-Ni_2P/NF. (b) The CV curve with low scan rate of Fe-CoP@Fe-Ni_2P/NF after activation.

=

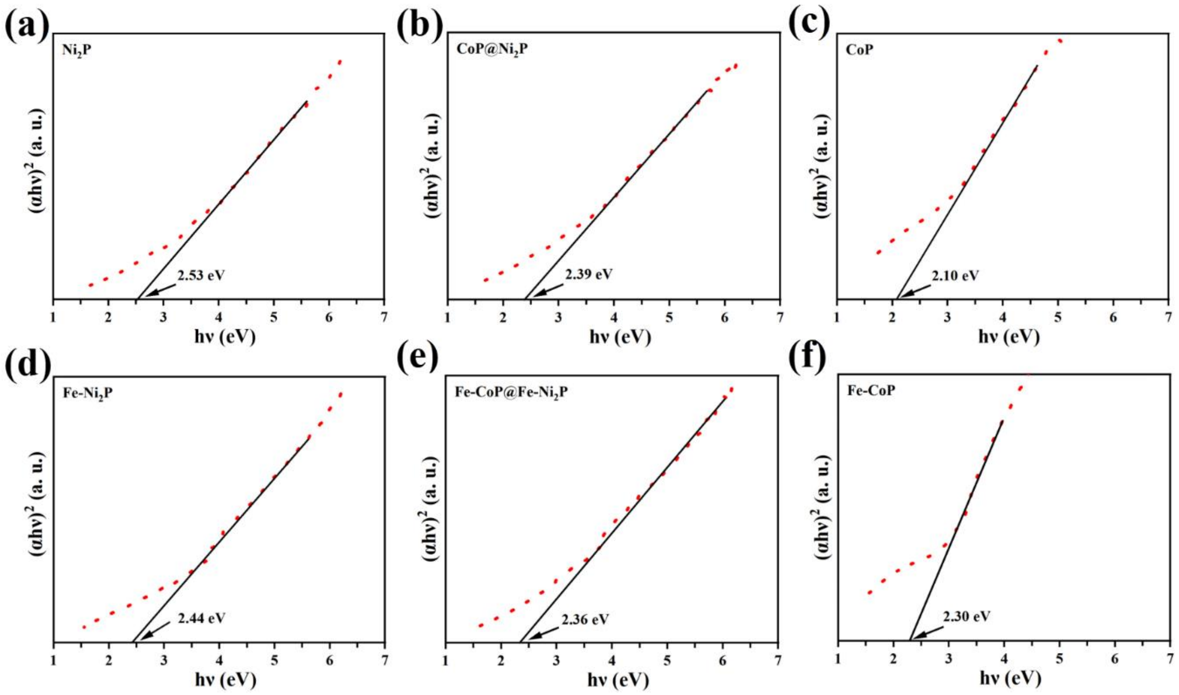

Figure S13. UV-vis spectra of (a) Ni_2P. (b) CoP@Ni_2P. (c) CoP. (d) Fe-Ni_2P. (e) Fe-CoP@Fe-Ni_2P. (f) Fe-CoP.

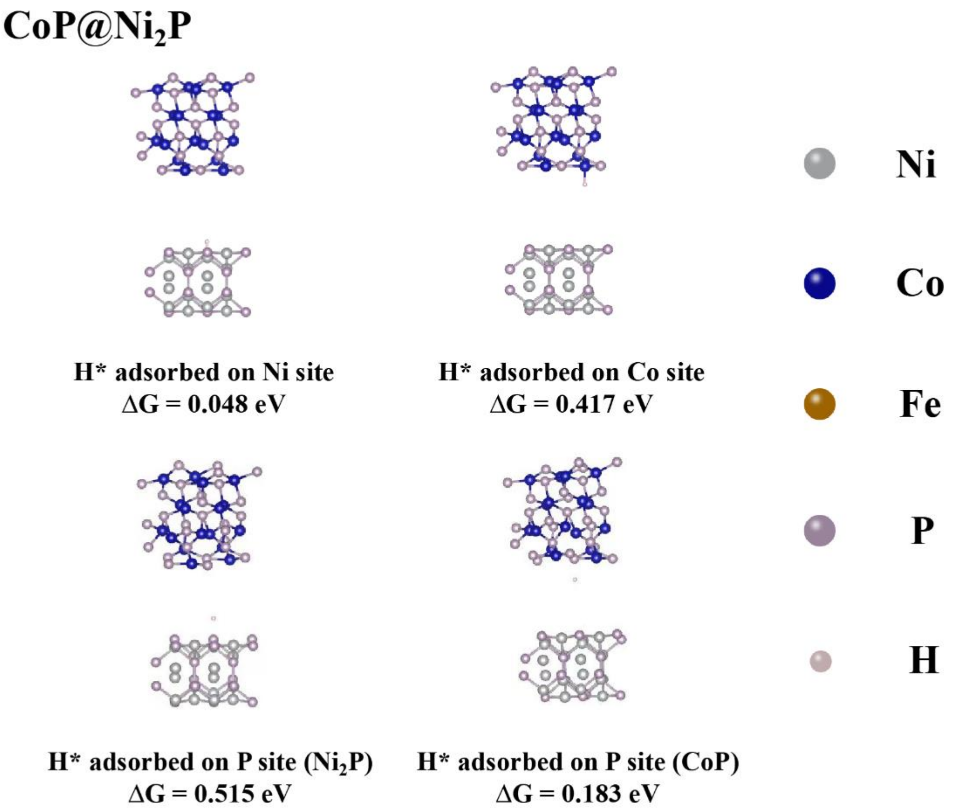

=

Figure S14. The HER reaction intermediates of $CoP@Ni_2P$ heterojunction on Ni, Co, P (Ni_2P) and P (CoP) sites.

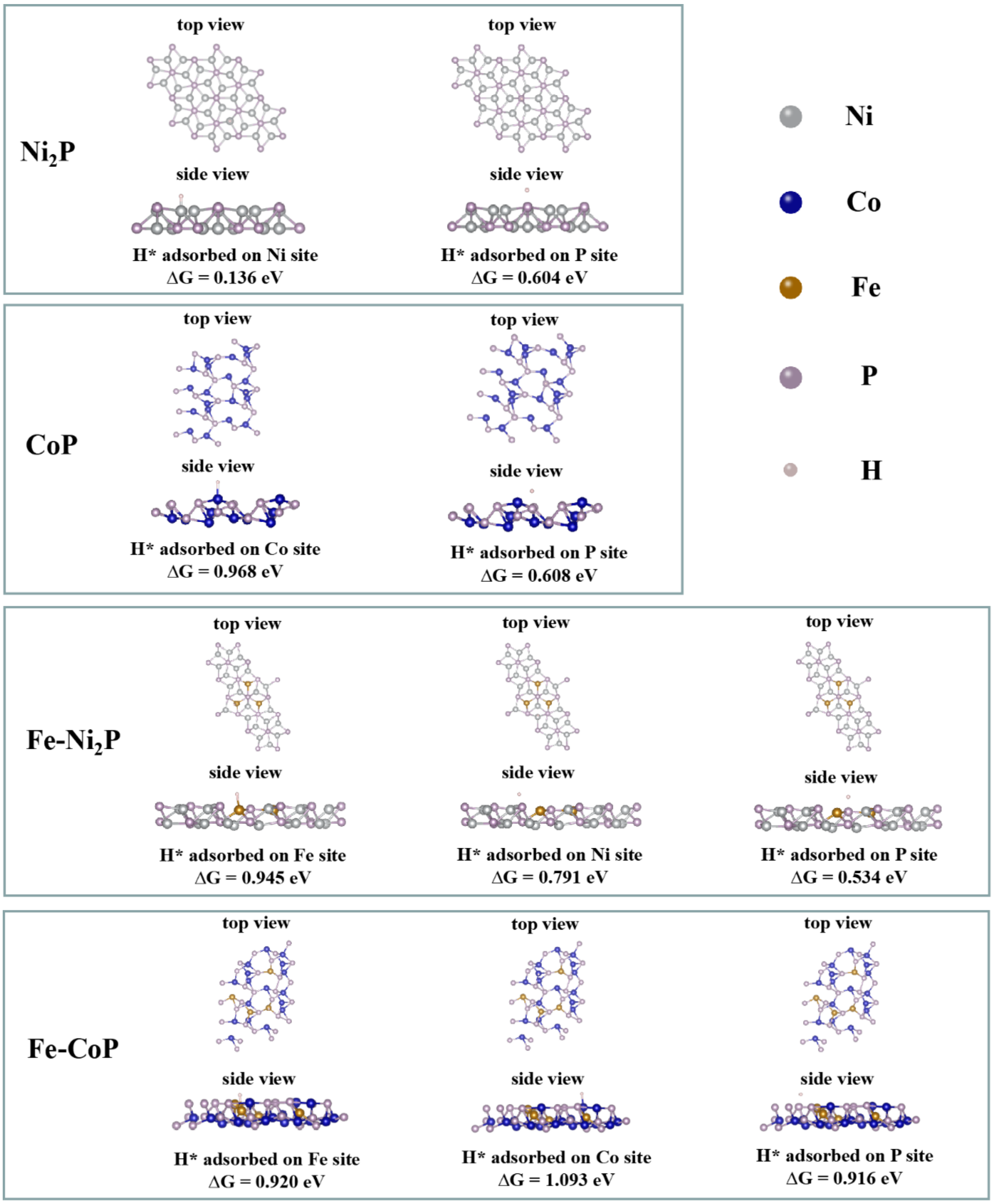

Figure S15. The HER reaction intermediates of Ni_2P, CoP, Fe-Ni_2P and Fe-CoP.

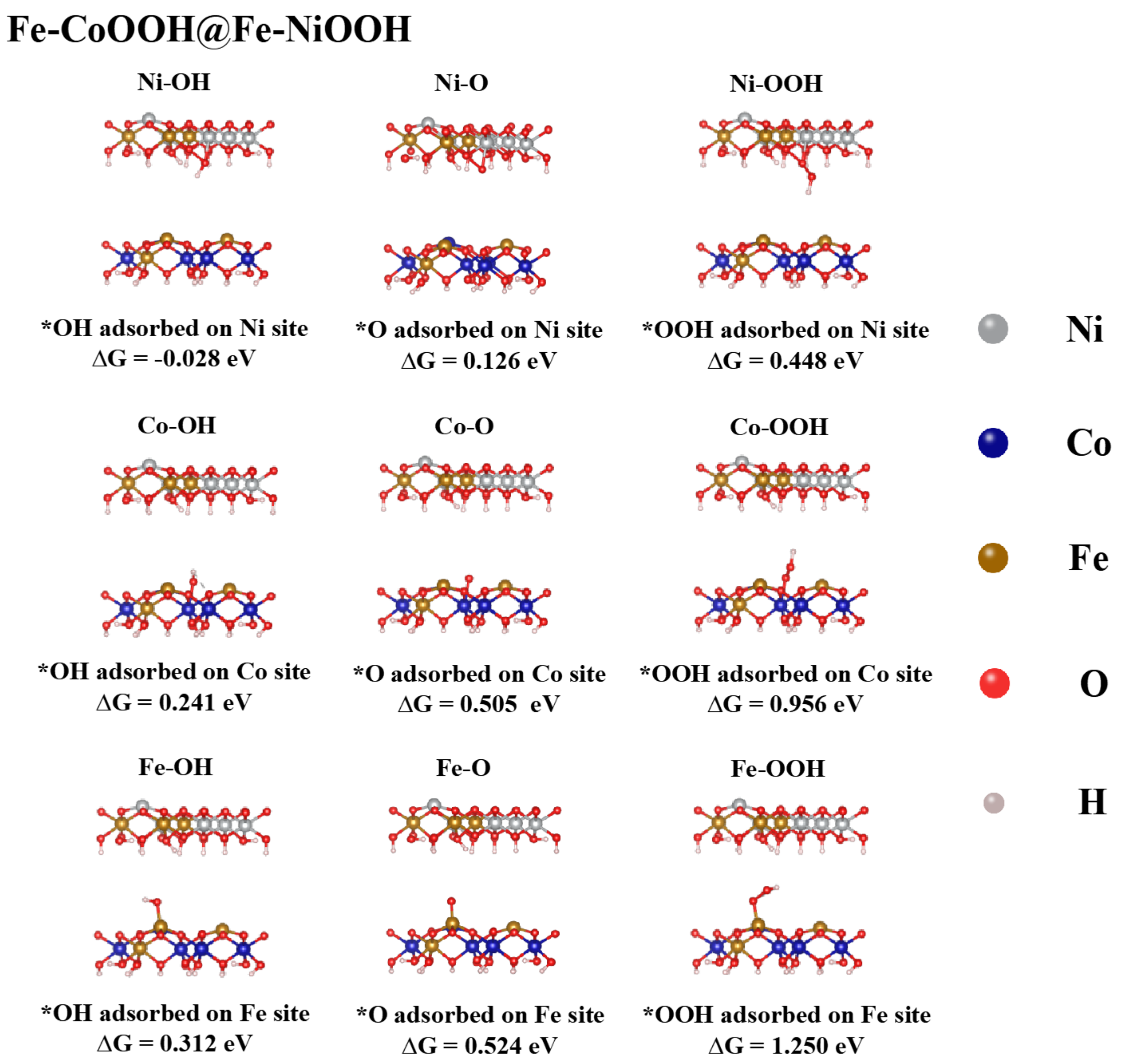

Figure S16. The OER reaction intermediates of Fe-CoOOH@Fe-NiOOH on Ni, Co and Fe sites.

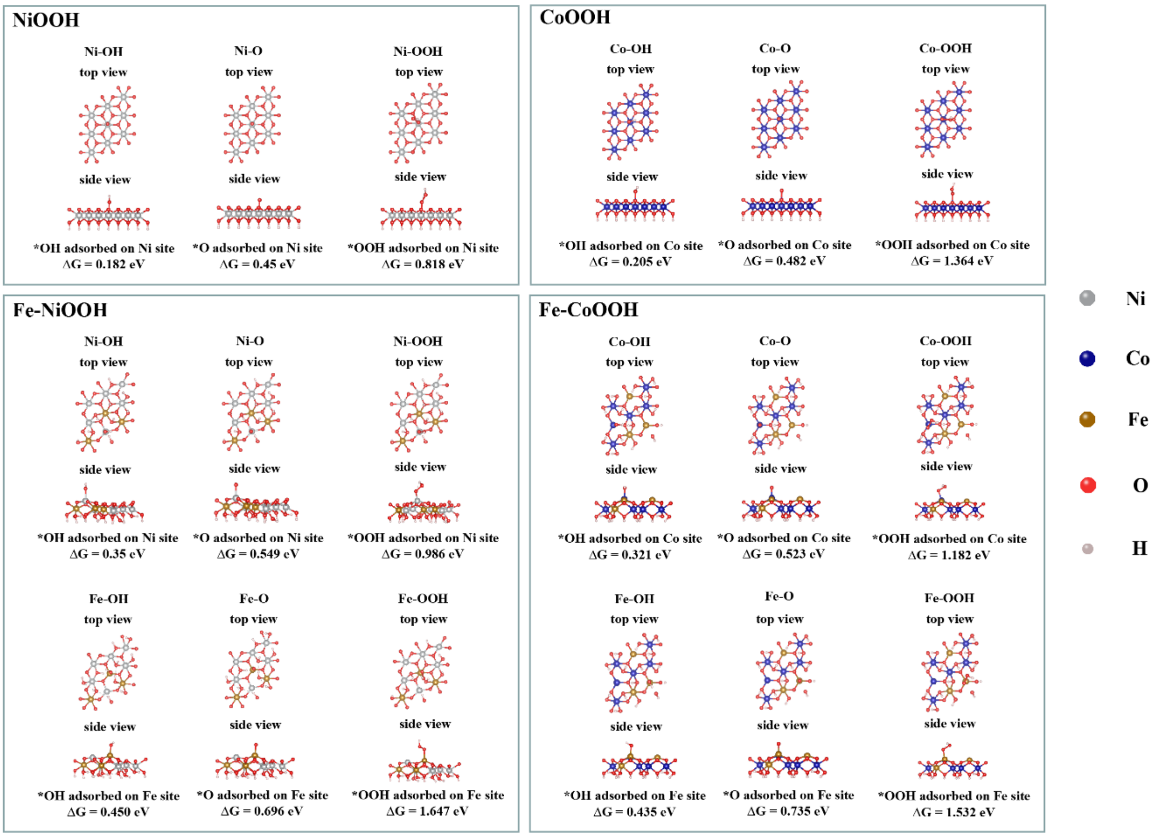

Figure S17. The OER reaction intermediates of NiOOH, CoOOH, Fe-NiOOH and Fe-CoOOH.

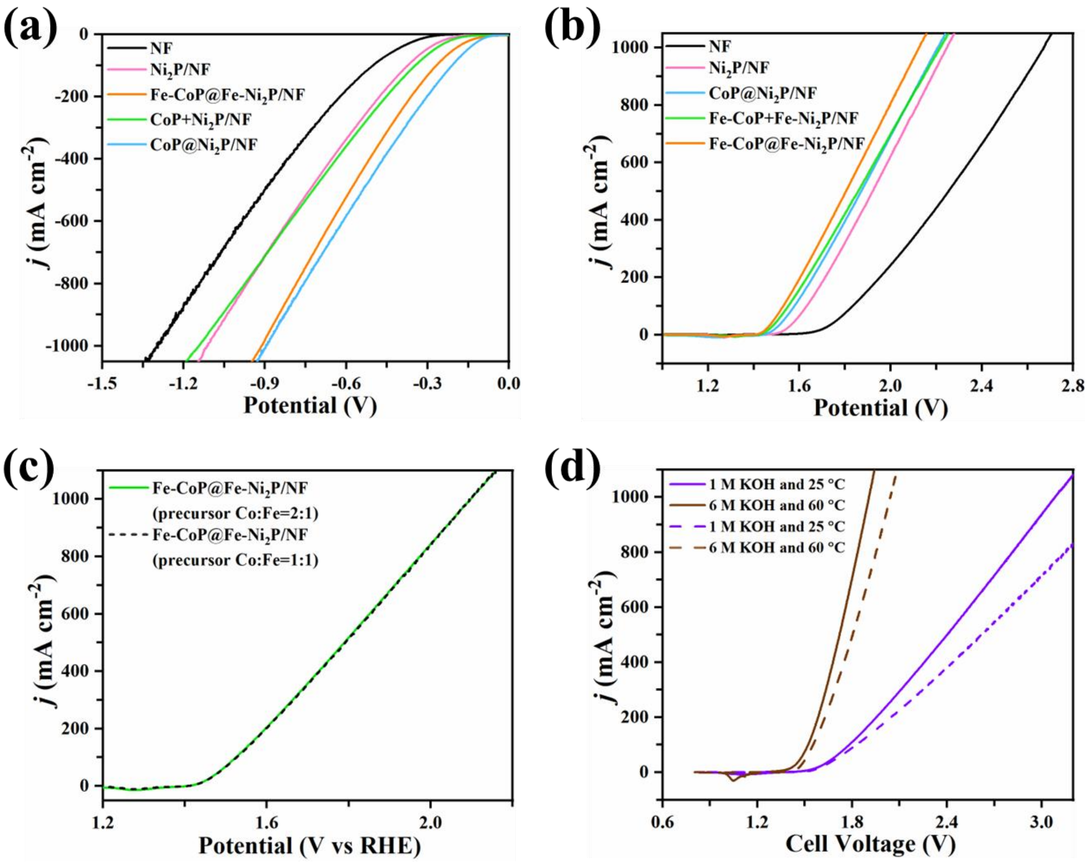

Figure S18. The Polarization curves of samples without iR compensation. (a) HER. (b) OER. (c) OER

of Fe-CoP@Fe-Ni_2P/NF prepared by increased molar ratio of Fe/Co precursors. (d) Overall water splitting (solid line: Fe-CoP@Fe-Ni_2P/NF (+) ‖ CoP@Ni_2P/NF (-), dotted line: RuO_2 (+) ‖ Pt/C (-)).

Table S1. Charge transfer resistances (R_{ct}) of samples for HER.

Catalysts	R_{ct} (Ω)
NF	262.80
Ni_2P/NF	23.69
CoP+Ni_2P/NF	14.61
CoP@Ni_2P/NF	1.52
Fe-CoP@Fe-Ni_2P/NF	3.69

Table S2. HER performances of different self-supported electrocatalysts in 1 M KOH electrolyte.

Samples	Overpotential (mV) j_{-1000}	Tafel slope (mV dec^{-1})	Support	Ref.
CoP@Ni_2P-NF	266	61.8	Ni Foam	This work
Fe-CoP@Fe-Ni_2P/NF	310	108	Ni Foam	This work
Co-Mo_5N_6/NF	280	27	Ni Foam	*Adv. Energy Mater.* **2020**, *10*, 2002176
IrNi-$FeNi_3$/NF	288.8	66.95	Ni Foam	*Appl. Catal. B: Environ.* **2021**, *286*, 119881
Porous Co-P/CF	290	78	Co Foam	*ACS Sustain. Chem. Eng.* **2020**, *8*, 10193
$Ni_{2(1-x)}Mo_{2x}P$ NWs/NF	294	46.4	Ni Foam	*Nano Energy* **2018**, *53*, 492
Nano-$K_2Fe_4O_7$/NF	302	47.1	Ni Foam	*J. Mater. Chem. A* **2021**, *9*, 7586
Ni_2P/NF	306	76	Ni Foam	*J. Am. Chem. Soc.* **2019**, *141*, 7537
N-NiMoS/NF	322	86	Ni Foam	*Appl. Catal. B: Environ.* **2020**, *276*, 119137
Ni@C-V_2O_3/NF	355	58.1	Ni Foam	*ACS Appl. Mater. Inter.* **2020**, *12*, 38061
CoS@NiFe LDH/NF	375	49	Ni Foam	*Small* **2022**, *18*, 2200586
A-NiCo LDH/NF	381	57	Ni Foam	*Appl. Catal. B: Environ.* **2020**, *261*, 118240

Table S3. Charge transfer resistances (R_{ct}) of samples for OER.

Catalysts	R_{ct} (Ω)
NF	674.5
Ni_2P/NF	9.95
Fe-CoP+Fe-Ni_2P/NF	1.55
CoP@Ni_2P/NF	2.38

Fe-CoP@Fe-Ni_2P/NF	1.07

Table S4. OER performances of different electrocatalysts in 1 M KOH electrolyte.

Samples	Overpotential η (mV) j_{1000}	Tafel slope (mV dec^{-1})	Support	Stability	Ref.
CoP@Ni_2P-NF	274	28.1	Ni Foam	-	This work
Fe-CoP@Fe-Ni_2P/NF	337	38.8	Ni Foam	Chronopotentiometry j = 1000 mA cm^{-2} 150 h; Δη=20 mV	This work
Ag/NiFe LDH/NF	276	23	Ni Foam	Chronopotentiometry j = 500 mA cm^{-2} 200 h; Δη=4.6 mV	*Nano Energy* **2022**, *98*, 107212
(Ni,Fe)OOH/NF	289	41.5	Ni Foam	Chronopotentiometry j = 1000 mA cm^{-2} 44 h; Δη=59 mV	*Energ. Environ. Sci.* **2018**, *11*, 2858
Ni_3S_2/Fe-NiP_x/NF	291	46.5	Ni Foam	Chronoamperometry j < 300 mA cm^{-2} 220 h; Δj≈8 mA cm^{-2}	*Adv. Sci.* **2022**, *9*, 2104846
FeP/Ni_2P/NF	293	22.7	Ni Foam	Chronopotentiometry j = 100 mA cm^{-2} 24 h; Δη≈0 mV	*Nat. Commun.* **2018**, *9*, 2551
$FeWO_4$-Ni_3S_2@C/NF	320	39.4	Ni Foam	Chronopotentiometry j = 1000 mA cm^{-2} 100 h; Δη=80 mV	*Chem. Eng. J.* **2022**, *434*, 134669
NiFe LDH/NiS/NF	325	60.1	Ni Foam	-	*Adv. Energy Mater.* **2021**, *11*, 2102353
Zn-(Ni/FeOOH)/NF	330	33	Ni Foam	Chronopotentiometry j = 1000 mA cm^{-2} 1000 h; Δη=55 mV	*Small* **2022**, *18*, 2203710
$NiMoO_x$/NiMoS/NF	334	34	Ni Foam	Chronoamperometry j ≤ 500 mA cm^{-2} 50 h; Δj≈10 mA cm^{-2}	*Nat. Commun.* **2020**, *11*, 5462
Ni_2P-Fe_2P/NF	337	58	Ni Foam	-	*Adv. Funct. Mater.* **2021**, *31*, 2006484
S-$FeOOH_{+1000}$/IF	358	50.8	Fe Foam	Chronopotentiometry j = 1000 mA cm^{-2} 1000 h; Δη≈20 mV	*Appl. Catal. B: Environ.* **2022**, *315*, 121571
NiMoN@NiFeN/NF	360	58.6	Ni Foam	Chronoamperometry j ≤ 500 mA cm^{-2} 48 h; Δj=18.6 mA cm^{-2}	*Nat. Commun.* **2019**, *10*, 5106
nano-$K_2Fe_4O_7$/NF	375	59	Ni Foam	Chronoamperometry j ≈ 1500 mA cm^{-2} 48 h; Δj≈0 mA cm^{-2}	*J. Mater. Chem. A* **2021**, *9*, 7586

Table S5. Faraday efficiencies of different electrocatalysts.

Samples	HER	OER	Support	Ref.
CoP@Ni_2P/NF	98.5%	-	Ni Foam	This work
Fe-CoP@Fe-Ni_2P/NF	-	97.2%		
NiCoVP	98%	-	Ni Foam	*J. Mater. Chem. A* **2021**, *9*, 12203
NiFeVP	-	95%		
$V_{0.3}$-NiS/NiS_2	≈98%		Graphene-coated Ni foam	*Adv. Energy Mater.* **2023**, *13*, 2300978
NiFe-MS/MOF@NF	≈98%		Ni Foam	*Adv. Sci.* **2020**, *7*, 2001965
P-$CoNi_2S_4$/CC	98%	96%	Carbon cloth	*Appl. Surf. Sci.* **2021**, *554*, 149598
Cobalt doped NFA	-	98%	Ni Foam	*Adv. Funct. Mater.* **2021**, *31*, 2008118
Fe-MOF/FF-5	-	98%	Fe Foam	*J. Colloid. Interf. Sci.* **2023**, *650*, 1290
$FeCoNiS_x$/NF	-	98%	Ni Foam	*ChemSusChem* **2022**, *15*, e202200590
NFCL(70)-LDH	-	97.5%	Ni Foam	*Adv. Funct. Mater.* **2022,** *32*, 2202072
NiTe@RuO_2 (-) ‖ NiTe@NiFe-LDH (+)	97 %		Ni Foam	*Appl. Catal. B: Environ.* **2020**, *272*, 118988
NiFeP@NiP@NF	97 %		Ni Foam	*ACS Appl. Mater. Interfaces* **2021**, *13*, 23702
$NiFe_2O_4$/NiFe LDH	-	92.6%	Ni Foam	*ACS Appl. Mater. Interfaces* **2018**, *10*, 26283
CdS@Co_9S_8/Ni_3S_2	≈90%		Co/Ni Alloy Foam	*J. Mater. Chem. A* **2020**, *8*, 3083

Table S6. Water splitting performances of different electrocatalysts in 1 M KOH electrolyte.

Cells (+ ‖ -)	Potential (V) $\eta_{1000\ mA/cm^2}$	Stability	Support	Ref.
Fe-CoP@Fe-Ni_2P/NF ‖ CoP@Ni_2P/NF	1.724	1000 mA cm^{-2} 150 h Chronopotentiometry Voltage increased by ~6% (~100 mV)	Ni Foam	This work
$(NiCo)_3Ce_4$ SABM ‖ $(NiCo)_3Ce_4$ SABM	1.750	1000 mA cm^{-2} 55 h Chronopotentiometry	(+) Ni Foam (-) Carbon paper	*Adv. Mater.* **2021**, *33*,

		Voltage increased by ~7.5% (~145 mV)		2103812
$Ni_{0.8}Fe_{0.2}$-AHNA ‖ Ni nanowire array	1.760	500 mA cm^{-2} 24 h Chronopotentiometry Voltage increased by ~5.8% (~100 mV)	Ni Foam	*Energ. Environ. Sci.* **2020**, *13*, 86
$SrCo_{0.85}Fe_{0.1}P_{0.05}O_{3-\delta}$/NF ‖ $SrCo_{0.85}Fe_{0.1}P_{0.05}O_{3-\delta}$/NF	1.780	10 mA cm^{-2} 650 h Chronopotentiometry Voltage increased by ~3.2% (~55 mV)	Ni Foam	*Adv. Mater.* **2018**, *30*, 1804333
FeP/Ni_2P/NF ‖ FeP/Ni_2P/NF	1.780	500 mA cm^{-2} 40 h Chronopotentiometry Voltage increased by ~1.5% (~25 mV)	Ni Foam	*Nat. Commun.* **2018**, *9*, 2551
Polarized NP-NiCoFeMoMn HEA ‖ NP-NiCoFeMoMn HEA	1.800	10 mA cm^{-2} 375 h Chronopotentiometry Voltage increased by ~1.6% (~23 mV)	Alloy ribbon	*Chem. Eng. J.* **2022**, *435*, 134898
NiFe-LDH/IF ‖ CoFeOH/CoFeP/IF	1.810	1000 mA cm^{-2} 1 h Chronopotentiometry no voltage increase	Fe Foam	*J. Power Sources* **2021**, *507*, 230279
$NiMoO_x$/NiMoS/NF ‖ $NiMoO_x$/NiMoS/NF	1.820	1.75 V 500 h (~500 mA cm^{-2}) Chronoamperometry no current density decrease	Ni Foam	*Nat. Commun.* **2020**, *11*, 5462
MnO_x/NiFeP/NF ‖ MnO_x/NiFeP/NF	1.828	500 mA cm^{-2} 120 h Chronopotentiometry Voltage increased by ~2.3% (~41 mV)	Ni Foam	*Small* **2022**, *18*, 2105803
(WO_2-$Ni_{17}W_3$)/$NiFe(OH)_x$/NF ‖ (WO_2-$Ni_{17}W_3$)/$NiFe(OH)_x$/NF	1.840	-	Ni Foam	*Chem. Eng. J.* **2022**, *431*, 134247
Mo-/Co-N-C/Cu ‖ Mo-/Co-N-C/Cu	1.908	1.6 V 80 h (~50 mA cm^{-2}) Chronoamperometry Current density decreased by ~21% (~12 mA cm^{-2})	Cu Scaffold	*Adv. Funct. Mater.* **2021**, *31*, 2102285
$NiCo_{(nf)}$-P ‖ $NiCo_{(nf)}$-P	1.940	1000 mA cm^{-2} 30 h Chronopotentiometry Voltage increased by ~3.2% (~62 mV)	Ni Foam	*ACS Sustain. Chem. Eng.* **2022**, *10*, 11577

Ni_2P-Fe_2P/NF ‖ Ni_2P-Fe_2P/NF	1.980	500 mA cm^{-2} 42 h Chronopotentiometry Voltage increased by ~3.9% (~72 mV)	Ni Foam	*Adv. Funct. Mater.* **2021**, *31*, 2006484
Fe-FVO-60-act ‖ Fe-FVO-60-act	2.000	100 mA cm^{-2} 85 h Chronopotentiometry Voltage increased by ~2% (~34 mV)	Ni Foam	*Chem. Eng. J.* **2021**, *416*, 129165
Ni/MoO_2@CN ‖ Ni/MoO_2@CN	2.020	1000 mA cm^{-2} 300 h Chronopotentiometry Voltage increased by ~2.9% (~60 mV)	Ni Foam	*Nano-Micro Lett.* **2022**, *14*, 20
$Ni_xFe_yMo_z$ LDH/NF ‖ $Ni_xFe_yMo_z$ LDH/NF	2.100	1000 mA cm^{-2} 20 h Chronopotentiometry no voltage increase	Ni Foam	*J. Mater. Chem. A* **2022**, *10*, 20497
NiFe LDH/NiS/NF ‖ NF	2.270	400 mA cm^{-2} 100 h Chronopotentiometry Voltage increased by ~0.9% (~13 mV)	Ni Foam	*Adv. Energy Mater.* **2021**, *11*, 2102353
Co_4N-CeO_2/GP ‖ Co_4N-CeO_2/GP	2.300	2.28 V 50 h (~1000 mA cm^{-2}) Chronoamperometry Current density decreased by ~10.4% (~104 mA cm^{-2})	Graphite Plate	*Adv. Funct. Mater.* **2020**, *30*, 1910596

Table S7. Water splitting performances of different electrocatalysts in 6 M KOH electrolyte at 60 °C.

Cells (+ ‖ -)	Potential (V) $\eta_{1000\ mA/cm^2}$	Stability	Support	Ref.
Fe-CoP@Fe-Ni_2P/NF ‖ CoP@Ni_2P/NF	1.620	1000 mA cm^{-2} 150 h Chronopotentiometry Voltage increased by ~12.6% (~210 mV)	Ni Foam	This work
$NiMoO_x$/NiMoS ‖ $NiMoO_x$/NiMoS	1.660	-	Ni Foam	*Nat. Commun.* **2020**, *11*, 5462
Ir/CoNiB ‖ Ir/CoNiB	1.720	-	Ni Foam	*Angew. Chem. Inter. Ed.* **2021**, *60*, 27126
Fe-Ni-O-N ‖ Ru/Fe-Ni-O-N	1.750	12 h (~500 mA cm^{-2}) Chronoamperometry no current density decrease	Ni-Fe Foam	*J. Mater. Chem. A* **2023**, *11*, 1886
NFCL(70)-LDH@NF	1.800	-	Ni Foam	*Adv. Funct.*

∥ NFL-LDH@NF				*Mater.* **2022**, *32*, 2202072
Ni/MoO_2@CN ∥ Ni/MoO_2@CN	1.860	1000 mA cm^{-2} 330 h Chronopotentiometry Voltage increased by ~4.2% (~80 mV)	Ni Foam	*Nano-Micro Lett.* **2022**, *14*, 20
SU-NiFe-LDH(TA) @NF ∥ NF	1.900	1000 mA cm^{-2} 800 h Chronopotentiometry no voltage increase	Ni Foam	*Nat. Commun.* **2023**, *14*, 6714
$FeWO_4$-Ni_3S_2@C/NF ∥ $FeWO_4$-Ni_3S_2@C/NF	1.940	1000 mA cm^{-2} 100 h Chronopotentiometry Voltage increased by ~9.3% (~180 mV)	Ni Foam	*Chem. Eng. J.* **2022**, *434*, 134669
(WO_2-$Ni_{17}W_3$)/NiFe$(OH)_x$ ∥ (WO_2-$Ni_{17}W_3$)/NiFe$(OH)_x$	2.100	1000 mA cm^{-2} 120 h Chronopotentiometry Voltage increased by ~4% (~84 mV)	Ni Foam	*Chem. Eng. J.* **2022**, *431*, 134247
F-Co_2P/NF ∥ F-Co_2P/NF	2.130	1000 mA cm^{-2} 50 h Chronopotentiometry Voltage increased by ~7.9% (~170 mV)	Ni Foam	*J. Mater. Chem. A* **2021**, *9*, 22626

Table S8. R_s of samples for iR compensation.

Samples	R_S (Ω)	
	HER	OER
NF	2.917	2.992
Ni_2P/NF	2.456	2.471
CoP+Ni_2P/NF	3.145	—
Fe-CoP+Fe-Ni_2P/NF	—	2.718
CoP@Ni_2P/NF	2.519	2.556
Fe-CoP@Fe-Ni_2P/NF	2.409	2.482
Fe-CoP@Fe-Ni_2P/NF(precursor Co:Fe=2:1)	—	2.310
Fe-CoP@Fe-Ni_2P/NF(precursor Co:Fe=1:1)	—	2.294
Overall water splitting (1 M KOH, room temperature) Fe-CoP@Fe-Ni_2P/NF (+) ∥ CoP@Ni_2P-NF (-)	5.432	
Overall water splitting (6 M KOH, 60 °C) Fe-CoP@Fe-Ni_2P/NF (+) ∥ CoP@Ni_2P-NF (-)	1.148	
Overall water splitting (1 M KOH, room temperature) RuO_2/NF (+) ∥ Pt/C/NF (-)	5.982	
Overall water splitting (6 M KOH, 60 °C) RuO_2/NF (+) ∥ Pt/C/NF (-)	1.342	
CoP@Ni_2P/NF (chronopotentiometric curve)	2.240 (0-12 h)<br>2.443 (12-24 h)<br>2.217 (24-36 h)	2.190 (84-96 h)<br>2.200 (96-108 h)<br>2.320 (108-120 h)

	2.282 (36-48 h)<br>2.252 (48-60 h)<br>2.205 (60-72 h)<br>2.417 (72-84 h)	2.400 (120-132 h)<br>2.295 (132-144 h)<br>2.175 (144-150 h)
Fe-CoP@Fe-Ni$_2$P/NF<br>(chronopotentiometric curve)	2.286 (0-12 h)<br>2.241 (12-24 h)<br>2.405 (24-36 h)<br>2.565 (36-48 h)<br>2.618 (48-60 h)<br>2.202 (60-72 h)<br>2.156 (72-84 h)	2.214 (84-96 h)<br>2.122 (96-108 h)<br>2.037 (108-120 h)<br>2.011 (120-132 h)<br>1.937 (132-144 h)<br>1.843 (144-150 h)
Overall water splitting<br>(chronopotentiometric curve, 1 M KOH, room temperature)<br>Fe-CoP@Fe-Ni$_2$P/NF (+) ‖ CoP@Ni$_2$P/NF (-)	4.953 (0-12 h)<br>5.247 (12-24 h)<br>5.122 (24-36 h)<br>5.168 (36-48 h)<br>5.020 (48-60 h)<br>5.187 (60-72 h)<br>5.265 (72-84 h)	5.276 (84-96 h)<br>5.189 (96-108 h)<br>5.165 (108-120 h)<br>5.482 (120-132 h)<br>5.262 (132-144 h)<br>5.809 (144-150 h)
Overall water splitting<br>(6 M KOH, 60 °C)<br>Fe-CoP@Fe-Ni$_2$P/NF (+) ‖ CoP@Ni$_2$P/NF (-)	1.583 (0-12 h)<br>1.407 (12-24 h)<br>1.390 (24-36 h)<br>1.380 (36-48 h)<br>1.197 (48-60 h)<br>1.204 (60-72 h)<br>1.251 (72-84 h)	1.388 (84-96 h)<br>1.324 (96-108 h)<br>1.346 (108-120 h)<br>1.194 (120-132 h)<br>1.161 (132-144 h)<br>1.176 (144-150 h)
